# Emerging Resistance and Virulence Patterns in *Salmonella enterica*: Insights into Silver Nanoparticles as an Antimicrobial Strategy

**DOI:** 10.3390/antibiotics14010046

**Published:** 2025-01-07

**Authors:** Irina Gheorghe-Barbu, Ilda Czobor Barbu, Rareș-Ionuț Dragomir, Ioana Cristina Marinaș, Miruna Silvia Stan, Radu Pericleanu, Andreea Ștefania Dumbravă, Liviu-Iulian Rotaru, Simona Paraschiv, Leontina Mirela Bănică, Ionuț Pecete, Dan Oțelea, Violeta Corina Cristea, Mircea Ioan Popa, Marilena Monica Țânțu, Marius Surleac

**Affiliations:** 1 Department of Botany and Microbiology, Faculty of Biology, University of Bucharest, 060101 Bucharest, Romania; irina.gheorghe@bio.unibuc.ro (I.G.-B.); r.dragomir20@s.bio.unibuc.ro (R.-I.D.); pericleanuradu@gmail.com (R.P.); andreeadum29@gmail.com (A.Ș.D.); rotaru.liviu-iulian@s.bio.unibuc.ro (L.-I.R.); 2 The Research Institute of the University of Bucharest (ICUB), 050095 Bucharest, Romania; ioana.cristina.marinas@gmail.com (I.C.M.); miruna.stan@bio.unibuc.ro (M.S.S.); marius.surleac@gmail.com (M.S.); 3 Department of Biochemistry and Molecular Biology, Faculty of Biology, University of Bucharest, 050095 Bucharest, Romania; 4 National Institute for Infectious Diseases Prof. Dr. Matei Bals, 021105 Bucharest, Romania; simona.paraschiv@umfcd.ro (S.P.); leontina.banica@umfcd.ro (L.M.B.); dan.otelea@gmail.com (D.O.); 5 Faculty of Medicine, Carol Davila University of Medicine and Pharmacy, 020021 Bucharest, Romania; dr.violetacristea@gmail.com (V.C.C.); mircea.ioan.popa@gmail.com (M.I.P.); 6 Synevo Central Lab Romania, 021408 Bucharest, Romania; pecete.ionut@yahoo.ro; 7 Cantacuzino National Medical Military Institute for Research and Development, 050096 Bucharest, Romania; 8 National University of Science and Technology Politechnica of Bucharest, 060042 Bucharest, Romania; marilena.tantu@upb.ro; 9 Department of Medical Assistance and Physiotherapy, Faculty of Sciences, Physical Education and Informatics, University of Pitești, 110040 Pitesti, Romania

**Keywords:** salmonellosis, *Salmonella enterica* Typhimurium, paediatric patients, alternative antimicrobial solutions based on silver nanoparticles

## Abstract

Background/Objectives: This study aims to characterize antibiotic resistance (AR) and virulence markers in *Salmonella* spp. isolated from Romanian outpatients’ stool samples. Methods: In 2019, community-acquired *Salmonella* strains were collected and identified using MALDI-TOF mass spectrometry, antibiotic susceptibility profiles have been determined with the MicroScan system, and soluble virulence factors were evaluated using specific culture media, while biofilm formation was quantified in 96-well plates. Molecular analysis targeted resistance genes for β-lactams (e.g., *bla*_TEM_ and *bla*_SHV_); tetracyclines (e.g., *tet(A)*); sulphonamides; and quinolones, as well as virulence genes (e.g., *invA*, *spvC*, *pldA*, and *held*). Whole-genome sequencing (WGS) was performed on 19 selected isolates. A silver nanoparticles (AgNPsol) alternative to conventional antibiotics was tested for effectiveness against multidrug-resistant (MDR) isolates. Results: From the total of 309 *Salmonella* isolates (65.05% from children under 4 years of age) belonging to four subtypes and four serovars, 27.86% showed resistance to at least one antibiotic, most frequently to tetracycline, ampicillin, and piperacillin. The strains frequently expressed haemolysin (67%), aesculinase (65%), and gelatinase (62%). Resistance to trimethoprim-sulfamethoxazole was encoded by the *sul1* gene in 44.83% of the strains and to tetracyclines by the *tet(A)* gene (59.52%). The ESBL genes *bla*_TEM_, *bla*_SHV_, and *bla*_CTX-M_ were detected by PCR in 16.18%, 2.91%, and 0.65% of the strains, respectively. Additionally, 98.63% of the strains carried the *invA* marker, with notable positive associations between *bla*_SHV_, *qnrB*, and *sul1* with *spvC*. Conclusions: The present findings revealed significant patterns in *Salmonella* isolates, subtypes, serovars, AR, and virulence, emphasising the need for continuous surveillance of *Salmonella* infections. Additionally, the potential of AgNPs as an alternative treatment option was demonstrated, particularly for paediatric *S. enterica* infections.

## 1. Introduction

The rising incidence of salmonellosis poses significant challenges to the management of human foodborne illnesses, as highlighted by Ziyate et al. in 2016 [1]. In Europe, salmonellosis ranks as the second most commonly reported zoonotic infection, according to research by Schmid and Baumgartner in 2013. Non-typhoidal *Salmonella enterica* is identified as a primary bacterium responsible for causing acute gastroenteritis among both children and adults, as noted by Al-Rifai et al. in 2020 [2]. This underscores the ongoing public health concern surrounding non-typhoidal *Salmonella* as a major contributor to gastrointestinal diseases globally. Antibiotic resistance (AR) in *Salmonella* spp. has been reported to occur in response to the most important classes of antimicrobial agents administered in humans (European Food Safety Authority, EFSA 2020) [3]. Antibiotic resistance genes (ARGs) allow multidrug resistance (MDR) in *Salmonella* spp. [4] via horizontal gene transfer (HGT). The presence of integrons within ARG cassettes has also been associated with AR in *Salmonella* spp. [5]. A set of frequently occurring ARGs has been detected in Gram-negative bacteria, with each gene linked to resistance against a specific antimicrobial agent. Two genes that are typically acquired to enable quinolone resistance in *Salmonella* spp. are *qnrA* and *qnrB* [6]. AR to trimethoprim-sulfamethoxazole can be acquired through the *sul1* and *sul2* genes [7]. Four genes commonly found in *Salmonella* spp. strains that are associated with tetracycline resistance are *tetA*, *tetB*, *tetC*, and *tetD* [8]. Resistance to β-lactams can be developed through extended-spectrum β-lactamases (ESBL), which are encoded by the *bla*_TEM_, *bla*_SHV_, and *bla*_CTX-M_ genes [9]. In addition, resistance to carbapenems is acquired through carbapenemases, which are encoded by several different genes, including *bla*_VIM_, *bla*_IMP_, *bla*_NDM_, *bla*_OXA-48_, and *bla*_KPC_ [10].

Biofilm formation allows bacterial cells to survive in the presence of antibiotics [11]. Furthermore, biofilms can facilitate the transfer of genetic elements in order to adapt to different selective pressures [12]. Studies have demonstrated that *Salmonella* spp. cells possess the capability to form biofilms, a trait that significantly contributes to their AR [13].

Although ARGs and virulence factors (VFs) are often thought to be mutually exclusive due to the parsimonious nature of bacterial genomes, which prioritize effective survival mechanisms [14], though this trade-off is not always observed [15]. Increasing evidence suggests that ARGs and VFs are likely to co-occur in order to allow survival, as well as effective colonisation of the hosts [16]. Recent bioinformatic analyses suggested that mobile genetic elements (MGEs) are often located near both ARGs and VFs encoding genes, facilitating HGT [15]. HGT through MGEs has allowed the co-selection of ARGs and VFs in bacterial communities [17]. It is not clear yet if increased AR directly correlates with an increase in virulence markers (VMs) in *Salmonella* spp. [14]. Some studies indicate that AR may be associated with decreased VF levels [14], whereas other studies suggested that AR can increase the virulence potential in *Salmonella* spp. [18,19].

The pathogenicity of *Salmonella* infections is determined not only by the invading bacterium, but is also to the host’s susceptibility factors such as age (young children and elderly individuals are more susceptible due to immature or weakened immune systems); genetic (polymorphisms in immune response genes: e.g., *NOD2*, *TLR4*, *IL-10*, hereditary conditions like hemochromatosis, and HLA type variations); and environmental factors (malnutrition, co-infections: e.g., HIV, and poor hygiene and sanitation significantly increase susceptibility to *Salmonella* infections) [20,21,22,23].

The pathogenic potential of *Salmonella* species is associated with specific virulence genes, which are found either on the chromosome or plasmids [24]. Among the VFs linked to *Salmonella* are *spv* (Salmonella plasmid virulence), *invA* (invasion gene) [25], *pldA* [26], and *helD* [27]. Typically, *Salmonella* VFs are located within pathogenicity islands (PIs) on either the chromosome or plasmids [28]. Chromosomal virulence genes, such as *invA*, encode proteins that enhance *Salmonella*’s ability to invade epithelial cells, contributing to its pathogenicity and serving as biomarkers. Plasmid-encoded virulence genes, such as *spv*, are thought to facilitate the bacterium’s systemic spread and colonisation of deep tissues [24].

In 2019, the European Union (EU) documented 90,105 cases of human salmonellosis with a stable notification rate of 20.0 cases per 100,000 people across 28 member states, closely aligning with the rate from the previous year. Countries such as Romania, Cyprus, Greece, Ireland, Italy, and Portugal reported notably low incidence rates, with 7.1 or fewer cases per 100,000 population [29]. By 2022, the total reported cases in the EU decreased to 65,208 across 27 Member States, maintaining a notification rate of 15.3 cases per 100,000 population. The same countries, with the addition of Bulgaria and Latvia, continued to report the lowest rates, each with 6.1 or fewer cases per 100,000 population [30]. These suggest a consistent management and possibly effective control measures in these countries over the observed years. In both 2019 and 2022, *Salmonella enterica* serovar Typhimurium remained the second most prevalent serotype in human infections in Europe, following *S. enterica* serovar Enteritidis. *S.* Typhimurium accounted for 11.9% of the cases in 2019 and 12.1% in 2022, as reported by the European Food Safety Authority [29,30]. The observed decrease in salmonellosis cases in the European Union between 2019 and 2022 may indicate improvements in public health measures, food safety practices, and infection control. However, attributing the reduction solely to management efforts should be approached with caution, as other factors, such as variations in reporting systems, diagnostic practices, or underreporting, could have contributed to the decline. While countries like Romania, Cyprus, Greece, and others consistently reported low incidence rates, further investigation and corroborating data are essential to validate the effectiveness of the control measures in these regions. Additionally, the continued prevalence of *S.* Enteritidis and *S.* Typhimurium highlights the need for targeted strategies to address these dominant serotypes.

Nanoparticles (NPs) are considered as a potent alternative to conventional antibiotics, particularly in combating AR. These particles are applied in diverse sectors such as biomedicine, pharmaceutics, cosmetics, food processing, and textile coatings. Among them, silver nanoparticles (AgNPs) have garnered significant interest for their potent antibacterial properties, demonstrating effectiveness against a broad spectrum of pathogens and bacterial biofilms [31,32,33]. The antibacterial effectiveness of AgNPs depends on their physicochemical characteristics, such as particle size, shape, surface charge, surface features, and magnetic properties. These attributes facilitate the NPs’ physical interactions with bacterial cell surfaces, enhanced by their expansive surface area, which optimises microbial contact. Notably, Franci et al. (2015) [34] reported that AgNPs disrupt bacterial cell membrane integrity, resulting in enhanced permeability. Furthermore, Rai et al. (2009) [35] highlighted that AgNPs disrupt the bacterial respiratory chain, thereby impeding cellular function. Various other studies have elucidated multiple antibacterial mechanisms attributed to AgNPs. These mechanisms involve disrupting the bacterial cell wall, generating reactive oxygen species (ROS), releasing Ag^+^ ions, and interacting with bacterial DNA [36]. The primary antibacterial actions of AgNPs are the release of Ag^+^ ions and the intracellular deposition of the NPs [37]. Additionally, the bactericidal effectiveness of AgNPs largely stems from the inhibition of nucleic acid, protein, and cell wall synthesis, coupled with the disruption of microbial cells [38].

The present study aims to investigate the relationships between AR and virulence genes in *Salmonella* spp. isolated from outpatient stool samples in Bucharest, Romania, during 2019. Additionally, we explored alternative treatments using AgNPs for the salmonellosis control in Bucharest, the capital city of Romania. To achieve this, we conducted both phenotypic and molecular analyses on a collection of *Salmonella* spp. isolates, which exhibited a variety of AR profiles. Following this initial assay, we tested AgNP-based treatments on 19 *S. enterica* strains that were thoroughly characterised by whole-genome sequencing.

## 2. Results

### 2.1. Isolate Characterisation

**Figure 1:**
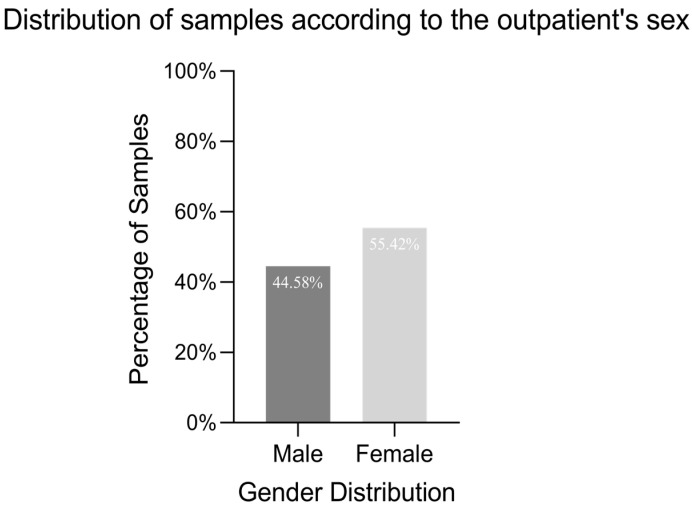
Distribution of samples according to the outpatient’s sex. Among the 309 isolates collected from faecal samples, 55.42% were derived from female patients, while 44.58% were isolated from male patients.

**Figure 2:**
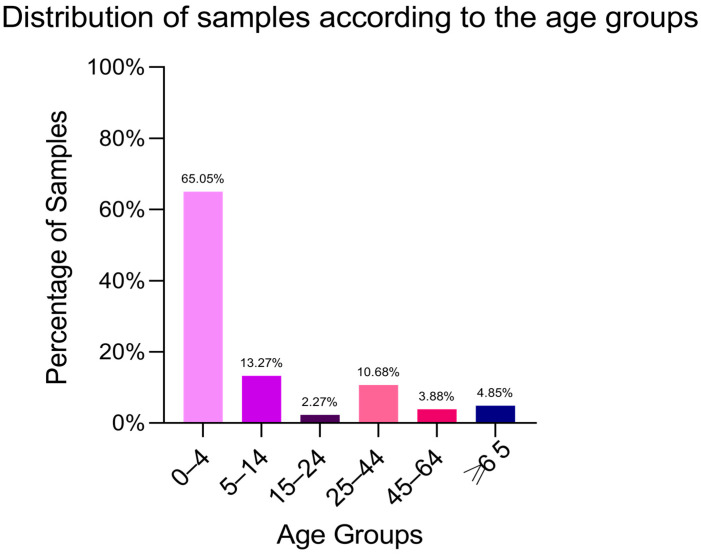
Distribution of samples according to the age groups. The majority of the 309 faecal samples were from children under 4 years old (65.05%), followed by decreasing frequencies in the age groups 5–14 years (13.27%), 25–44 years (10.68%), over 65 years (4.85%), 45–64 years (3.88%), and 15–24 years (2.27%).

Of the 309 isolates from faecal samples, 170 (55.42% of the isolates) were collected from female patients (Figure 1), and 65.05% of the isolates were recovered from children under 4 years old. The age distribution of the outpatients suggests that salmonellosis is more prevalent in children amongst individuals tested in one private laboratory setting in Bucharest. Two additional samples categories, ranked in descending order of frequency, correspond to the age intervals 5–14 years and 25–44 years, accounting for 13.27% and 10.68% of the total collected samples, respectively (Figure 2).

**Figure 3:**
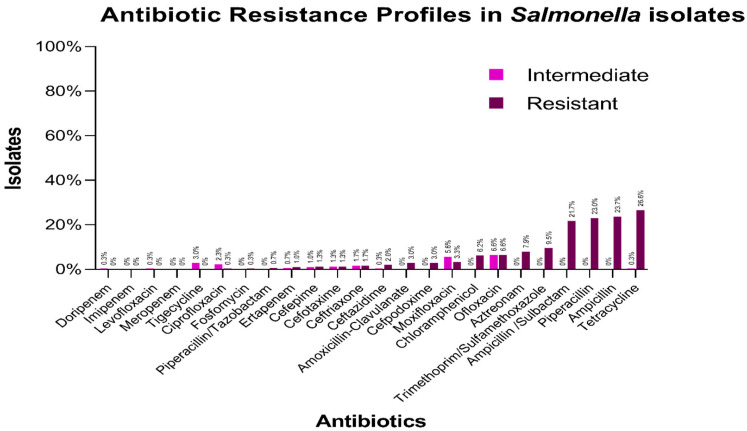
Resistance to different antibiotics in the sampled *Salmonella* isolates. The highest resistance rates correspond to Tetracyclines (26.6%), Ampicillin (23.7%), Piperacillin (23.0%), and the β-lactam inhibitor Ampicillin-Sulbactam (21.96%). Moderate levels of resistance were observed for Trimethoprim/Sulfamethoxazole (9.53%), Aztreonam (7.89%), Ofloxacin (6.55%), and Chloramphenicol (6.22%). Lower resistance rates were noted for Moxifloxacin (3.27%), Amoxicillin-Clavulanic Acid (2.95%), Cefpodoxime (2.96%), Ceftazidime (1.97%), Ceftriaxone (1.65%), Cefotaxime (1.32%), and Cefepime (1.31%). Notably, no resistance was detected for Imipenem, Meropenem, Tigecycline, or Levofloxacin, indicating the preserved efficacy of these antibiotics against the tested *Salmonella* strains.

The highest resistance levels were to Tetracyclines (26.55% with the minimum inhibitory concentration (MIC) values > 8 µg/mL), Ampicillin (23.68%, MIC > 8 µg/mL), and Piperacillin (23.02%, MIC > 16 µg/mL), along with the β-lactam inhibitor Ampicillin-Sulbactam (21.96%, MIC > 8/4 µg/mL). In decreasing order, it was found that 9.53% of the strains were resistant to Trimethoprim/Sulfamethoxazole (MIC > 4/76 µg/mL), 7.89% to Aztreonam (MIC = 16 µg/mL), 6.55% for Ofloxacin (MIC > 1 µg/mL), 6.22% to Chloramphenicol (MIC > 8 µg/mL), 3.27% to Moxifloxacin (MIC > 1 µg/mL), and 2.95% to Amoxicillin-Clavulanic Acid (MIC > 8/4 µg/mL) (Figure 3, Supplementary Table S2). The AR level to cephalosporins was low, the resistance level to Cefpodoxime, Ceftazidime, Ceftriaxone, Cefotaxime, and Cefepime being 2.96% (MIC > 1 µg/mL) > 1.97% (MIC > 16 µg/mL) > 1.65% (MIC = 2 µg/mL) > 1.32% (MIC = 16 µg/mL) > 1.31% (MIC ≥ 8 µg/mL). No resistance was found to Imipenem, Meropenem, Tigecycline, and Levofloxacin (Figure 3, Supplementary Table S4).

Additionally, 61 isolates were found to exhibit the MDR phenotype according to the antimicrobial agent categories for Enterobacteriaceae, set out by Magiorakos et al. (2012) [39].

### 2.2. Soluble Enzymatic VFs Detection

When evaluating for virulence factors, most of the isolates presented haemolytic (88% of the isolates, β and α haemolysins, pore-forming toxins (PFTs) that break down haemoglobin in red blood cells, leading to the release of haemoglobin into the surrounding medium) and esculin-associated (89%) activity that refers to the hydrolysis of the glycoside esculin into glucose and aesculetin, with the highest activity found in the 0–14 years age group of isolates. An important number of isolates were also positive for gelatinase, a proteolytic enzyme that degrades gelatine into amino acids (256/309), followed by lipase (lipids degradation into fatty acids and glycerol), caseinase (breaks down casein, the main protein found in milk and dairy products), amylase (starch degradation into simpler sugars such as maltose and glucose), and lecithinase (targets lecithin, a major component of cell membranes) (Table 1). As can be seen in Table 1, there is a correlation between young age and VFs (chi-square = 60.21; *p*-value = 0.0009).

### 2.3. Biofilm Formation

Most biofilm-forming isolates only showed a weak capacity, and only one isolate showed moderate capacity. Therefore, 6% of the tested *Salmonella* isolates displayed the ability to form biofilms. No association was found between the presence of ARGs and/or VFs and the biofilm formation ability (Supplementary Table S5).

### 2.4. AR and Virulence Genes

**Figure 4:**
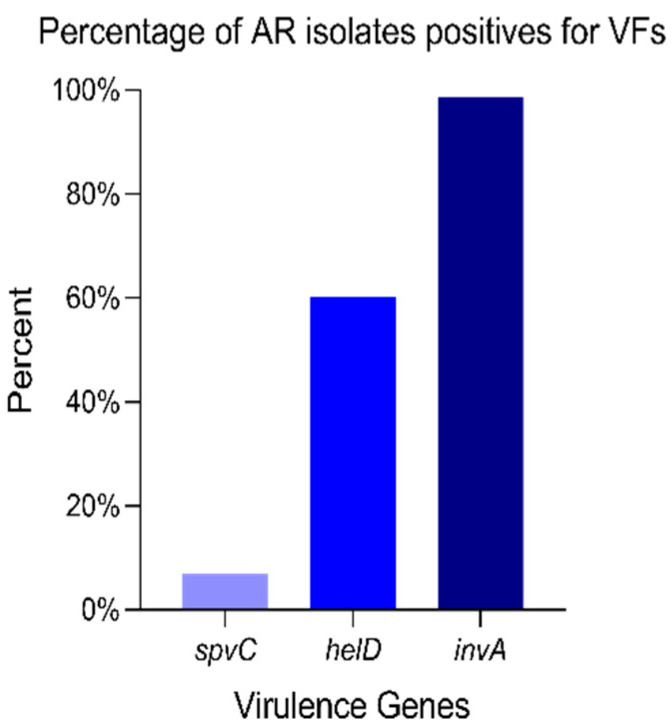
AR isolates positive for VFs. Among the 201 isolates resistant to at least one antibiotic class, 98.63% of isolates were positive for the *invA*, 60.27% for the *helD* gene, and 6.85% for the *spvC* gene.

Additionally, 44.83% and 17.24%, respectively, of the Trimethoprim-Sulfamethoxazole resistant isolates were positive for *sul1* and *sul2* genes (Figure 4), 59.52% of the Tetracycline resistant isolates possessed the *tetA* gene, and 2% the *tetD* gene (Figure 4), while *tetB* and *tetC* were absent in all investigated samples.

The plasmid-mediated quinolone resistance (PMQR) genes *qnrA* and *qnrB* were found in 1.62% and 2.6% of the tested samples, respectively.

**Figure 5:**
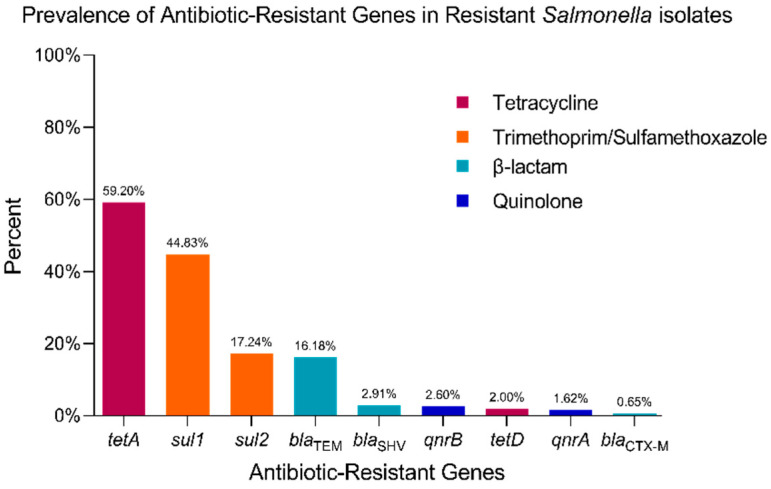
Prevalence of ARGs in resistant isolates. Among 84 Tetracycline-resistant strains, 59.20% carried the *tetA* gene, and 2% harboured the *tetD* gene. Among 29 Trimethoprim-Sulfamethoxazole-resistant strains, 44.83% were positive for the *sul1* gene and 17.24% for the *sul2* gene. For fluoroquinolone resistance, among 21 Moxifloxacin-resistant isolates, 9 Ciprofloxacin-resistant isolates, and 3 Levofloxacin-resistant isolates, *qnrA* and *qnrB* genes were identified in 1.62% and 2.6% of the samples, respectively. For ESBL strains: resistance to Ceftriaxone (10 isolates), Amoxicillin-Clavulanic Acid (9 isolates), Cefpodoxime (8 isolates), Cefotaxime (6 isolates), Ceftazidime (6 isolates), and Cefepime (4 isolates). ESBL-encoding genes were detected in 16.18% (*bla*_TEM_), 2.91% (*bla*_SHV_), and 0.65% (*bla*_CTX-M_) of the isolates.

The ESBL encoding genes *bla*_TEM_, *bla*_SHV_, and *bla*_CTX-M_ were present in 16.18%, 2.91%, and 0.65% of the samples (Figure 5), while *bla*_VIM_, *bla*_IMP_, *bla*_NDM_, *bla*_OXA-48_, and *bla*_KPC_ were not detected by PCR reaction.

It has been observed that the isolates that were resistant for at least one antibiotic class were positive for the *invA* VF (98.63%), *helD* (60.27%), and *spvC* (6.85%) genes (Figure 4).

The potential correlation between ARGs and VFs presence in the investigated isolates was evaluated using Fisher’s exact test. The ARGs *tetA*, *sul1*, *qnrB*, *bla*_TEM_, and *bla*_SHV_ were all found to be correlated with the presence of the *spvC* VF (Supplementary Table S6).

### 2.5. WGS Analysis of Salmonella enterica subsp. Enterica Isolates

The WGS analysis of 19 *S. enterica* isolates collected from paediatric patients (aged between 2 months and 6 years) revealed the presence of various ARGs. The majority of antibiotic resistance (AR) genes identified belong to the aminoglycoside, tetracycline, and sulfonamide classes, with the lowest prevalence observed in isolates S34, S58, and S93. Resistance to aminoglycosides is conferred by the presence of such genes as *aadA1*, *aadA2*, *aadA5*, *ant(2″)-Ia*, *aph(3″)-Ib*, and *aph(6)-Id*. Macrolide resistance is associated with the presence of the *mph(A)* gene, while Tetracycline resistance seems to be mediated by the *tet(A)* and *tet(B)* genes. Additionally, the isolates harboured the sulphonamide resistance genes *sul1*, *sul2*, and *sul3*. Genes encoding resistance to Chloramphenicol (*cmlA1* and *cmlA5*); trimethoprim (*dfrA5*, *dfrA12*, and *dfrA17*); and quinolones (*qnrA1* and *qnrB19*) were also detected. Furthermore, the *bla*_TEM-1_ gene, encoding a class A broad-spectrum β-lactamase, was identified in association with two distinct *S. enterica* clones (ST34 and ST19). These findings highlight the diversity of the AR determinants present in the analysed isolates (Table 2). The ESBL encoding genes *bla*_SHV_ and *bla*_CTX-M_ were not identified in analysed *Salmonella* isolates by WGS.

Conversely, biocide resistance (BR) genes were found at higher incidence, with most classified as metal resistance genes and efflux systems (see the Supplementary File S1).

The analysed strains were classified into four sequence types (STs), with the majority belonging to a widespread clone (ST19, n = 10) associated with MDR plasmid replicons, including those encoding TEM-1 β-lactamase ((IncB/O/K/Z_2, IncFIB(S)_1, IncFIB(AP001918)1, IncFII(S)_1, IncX3_1, IncX1_1, IncI1_1_Alpha, ColRNAI_1, Col440I_1, Col8282_1, and Col(VCM04)_1). Additionally, ST34 (n = 5 isolates), which is of significant public health concern, was identified, given its association with MDR and its potential to cause widespread outbreaks, particularly in Europe and Asia. Two relatively less common sequence types of *S. enterica* were also identified: ST33 (n = 3) and ST32 (n = 1) (Table 2).

The most frequently identified MGEs included (1) integrative conjugative elements (ICEs) like ICEVchBan*9* (harbouring *tet* and *sul* genes) [40] and ICEHin*1056* (a genomic island linked to antibiotic resistance, carrying β-lactamases in this study) [41]; (2) integrons (In), such as In*185*, In*221*, In*511*, In*705*, and In*784*; (3) insertion sequences (IS), including IS*200F* (specific to *S. enterica*) [42], IS*15DIV* (associated with *bla*_NDM5_) [43], and IS*Sen1*, which appeared frequently across most isolates; (4) genomic islands (GIs) like SGI*1* and SGI*2*; and (5) Transposons (Tn), including Tn*21*, Tn*6011*, Tn*6024*, and Tn*6090* (see Supplementary File S1).

The 19 *S. enterica* isolates belonged to four serovars: S. *enterica* Typhimurium (n = 10 isolates), linked to the widespread ST19; *S.* *enterica* 1,4,[5],12:i:-, the monophasic variant of the recognized pathogenic *S. enterica* Typhimurium (5 isolates) is linked to ST34; *S. enterica* Hadar identified in three isolates and that is associated with ST33; and *S. enterica* Infantis in one isolate that belongs to ST32 (Table 3).

Using the Virulence Factor Database (VFDB), the analysis of circulating *S. enterica* clones from outpatients in Bucharest, Romania, revealed the presence of several virulence factors associated with distinct pathogenic mechanisms. These factors were linked to functions such as bacterial adherence (*fimI*, *fimC*, *fimD*, *fimH*, *fimF*, *csgC*, *csgA*, *csgB*, *csgD*, *csgE*, *csgF*, *csgG*, *sinH*, *ratB*, *lpfE*, *lpfD*, *lpfC*, *lpfB*, *lpfA*, *misL*, and *ompA*); the delivery of effector proteins via type III secretion systems (*invH*, *invF*, *invG*, *invE*, *invA*, *invB*, *invC*, *invI*, *invJ*, *sopD*, *ssaU*, *ssaT*, *ssaS*, *ssaR*, *ssaQ*, *ssaP*, *ssaO*, *ssaN*, *ssaV*, *ssaM*, *ssaL*, *ssaK*, *ssaJ*, *ssaI*, *ssaH*, *ssaG*, *sseG*, *sseF*, *sscB*, *sseE*, *sseD*, *sseC*, *sscA*, *sseB*, *sseA*, *ssaE*, *ssaD*, *ssaC*, *avrA*, *sseL*, *sseK2*, *sseI/srfH*, *slrP*, *sseK1*, *sopD2*, *sopA*, *sopE2*, *steC*, *sseJ*, *steB*, *sifB*, *steA*, *sicA*, *sipB/sspB*, *sipC/sspC*, *sipD*, *sipA/sspA*, *sicP*, *gogB*, *spaO*, *spaP*, *spaQ*, *spaR*, *spaS*, *sopB/sigD*, *orgA*, *orgB*, *orgC*, *pipB*, *pipB2*, *prgH*, *prgI*, *prgJ*, *prgK*, *sptP*, *sifA*, *pipB2*, *spvC*, *B*, and *R*); immune system modulation (*rck*); and metabolic functions (*entB*, *entA*, *mgtB*, *mgtC*, *fepC*, and *fepG*). Additional virulence determinants were linked to stress response (*sodCI*), regulation of virulence gene expression (*spiC/ssaB*), and competitive advantages (*mig-14*). The clone associated with the highest number of virulence factors was ST19, belonging to *S.* Typhimurium, and was identified in 10 isolates, which were dispersed through various plasmid types (Supplementary Table S7).

### 2.6. Pangenome Analysis of S. enterica Strains

**Figure 6:**
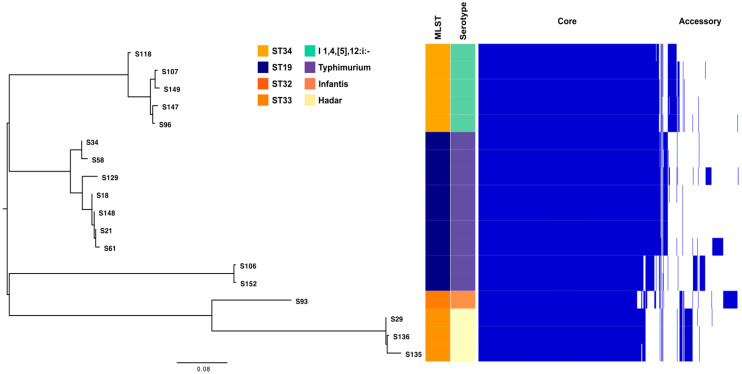
Pangenome tree for *S. enterica* isolates. Distinct phylogenetic clusters of *Salmonella* isolates were identified based on their STs and serotypes. Cluster 1 comprised *S.* Hadar isolates associated with ST33, while Cluster 2 included isolates linked to ST34 are linked to the monophasic variant of the *S.* Typhimurium serotype. Additional clusters were formed by isolates associated with ST19, indicative of the *S.* Typhimurium serotype. A single isolate, encoded S93, associated with ST32 and *S.* Infantis serotype, remained ungrouped due to its unique sequence type and serotype, highlighting its distinct genetic and phenotypic characteristics.

The analysis revealed distinct clusters, each associated with specific STs and serotypes: cluster 1 (highlighted in light yellow in Figure 6) encompasses isolates of *S.* Hadar serotype, all of which are linked to ST33; cluster 2 (represented in mint green) includes isolates associated with ST34, which corresponds to the monophasic variant of *S.* Typhimurium serotype. Other clusters represent isolates belonging to ST19, indicative of *S.* Typhimurium serotype. Isolate S93 (linked to ST32 and the Infantis serotype) did not group with other clusters, aligning with its unique ST and serotype characteristics.

In accordance with Heaps’ law, the pangenome of *S. enterica* remains open, characterized by a γ-value of 0.11. This open nature suggests the continuous acquisition of new genes across isolates, reinforcing the genetic diversity within *S. enterica*.

### 2.7. AMR, MGE and Phage Predictions in Context of Clustering

Overall, approximately 2600 gene clusters were identified across the 19 isolates. Of these, around 1500 (not exactly clusters) are individual genes, while the remaining form clusters of two and up to 45 elements, with cluster sizes extending up to 40 kb. Clusters were categorized based on length, with multiple clusters often found within a single genome. About 2000 clusters are up to 5 kb in length, containing between one and seven elements, including most AR and BR genes, and MGEs like IS, integrative-conjugative elements (ICE), as well as toxin/antitoxin systems. AR genes typically appear alone on contigs, while BR genes range from solitary to clusters of three elements. Larger clusters in this range mainly consist of virulence factors. A smaller set of 330 clusters ranges from 5 to 10 kb, predominantly consisting of BR, VF, and phage elements. The 10–15 kb cluster set includes approximately 160 clusters, comprising VF, phages, ICEs, and elements linked to pathogenicity islands (PAIs). Some elements are classified as genomic islands (GIs) (e.g., resistance islands (RI) or PAIMGE when multiple elements are predicted to be in the same region by different predictors. Some VF clusters are flanked by BR genes. The 15–20 kb set contains 23 clusters, mostly composed of phage or PAI elements flanked by toxin/antitoxin systems.

Additionally, 70 clusters exceed 20 kb in length, and these are primarily made of phage elements. These phage clusters, in the last set, divide into two size groups (~21 kb and ~39 kb). Smaller phage clusters are typically flanked by a *tnpA* transposase and contain a central VF, *sodCI*. All phage clusters in this set were unique to *S.* Typhimurium ST19 isolates. Additionally, clusters between 25 and 33 kb were observed, consisting of virulence factors and pathogenicity island elements. Some longer clusters were flanked by toxin/antitoxin systems, while others comprised only VF (e.g., a 25 kb VF cluster) or PAI elements alone, such as a 21 kb cluster containing *hlyD* (a hemolysin secretion protein involved in RTX toxin secretion in *E. coli*). These findings indicate potential pathogenicity islands circulating among *S. enterica* isolates from various serotypes (see Supplementary File S1).

### 2.8. Conjugation Experiments

Conjugation experiments revealed that the conjugation frequency (Cf) varied depending on the donor strain, as shown in Table 4. For conjugations using *Salmonella* Hadar strains associated with ST33 as donors, the conjugation frequency ranged from 2.36 × 10^−10^ to 3.3 × 10^−6^. In contrast, experiments using *Salmonella* Typhimurium monophasic variant strains linked to ST34 yielded a higher frequency, between 3.33 × 10^−1^ and 3.5. Furthermore, PCR amplification targeting the *tet(A)* and *tet(B)* genes showed that these resistance determinants were absent in the resulting transconjugants.

### 2.9. Antimicrobial Efficiency of AgNPs Against S. Typhimurium, S. Hadar and S. Infantis

#### 2.9.1. Qualitative Screening of AgNP Against Selected *S. enterica* Isolates

**Figure 7:**
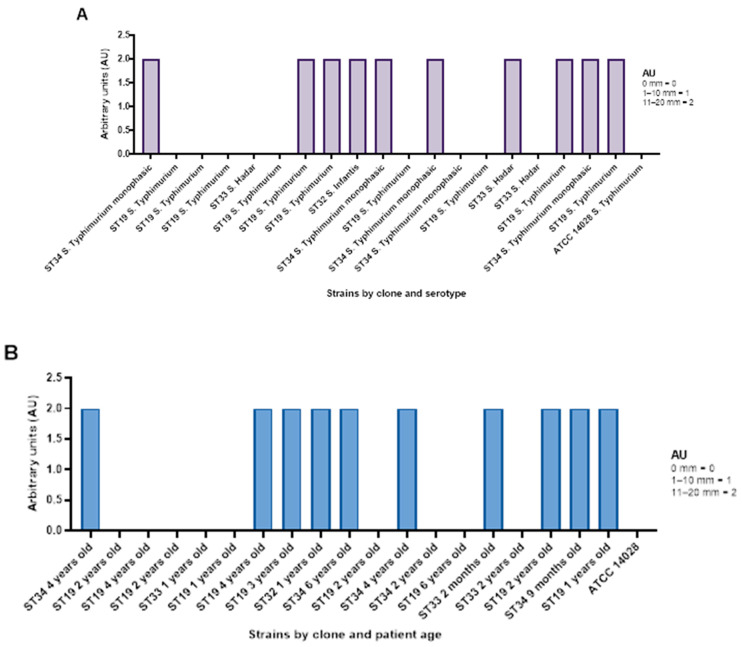
The diameter of the inhibition zone converted into arbitrary units of *S. enterica* isolates sorted by clone and serotype (**A**) and by clone and patient age (**B**).

Out of 19 *S. enterica* isolates, along with *S. enterica* ATCC 14028, AgNPsol demonstrated efficiency in 50% of cases, indicated by an arbitrary unit (AU) 2. When analysing the efficiency of AgNPsol by serovar, the highest activity was observed against the *S.* Infantis (100%) > S 4,[5],12:i:- belonging to ST34 (80% of the isolates) > *S.* Hadar ST33 (33% of the isolates) > *S.* Typhimurium ST19 (30%). In the case of 58% of the ambulatory isolates, the qualitative screening revealed that AgNPsol was ineffective (AU = 0) (Figure 7A, Supplementary Table S8).

The antimicrobial efficiency of AgNPsol against *S. enterica* was demonstrated in paediatric patients aged 2 months to 6 years, with an AU = 2 in 50% of the cases. This suggests that AgNPsol exhibits a measurable antibacterial effect in this age group, indicating its potential as a therapeutic alternative for treating *S. enterica* infections, particularly in younger patients (Figure 7B).

AgNP synthetised by classical method (encoded Ag2NP) was inefficient to combat the *S. enterica* infections in paediatric patients (see Supplementary Figure S9).

#### 2.9.2. Quantitative Antibacterial Evaluation of AgNPsol Against Selected *S. enterica* Isolates

**Figure 8:**
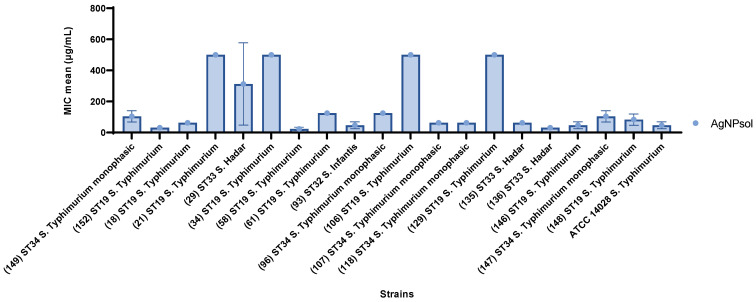
Graphical representation of the MIC values determined by microdilution method of AgNPsol against *S. enterica* isolates (n = 3 replicates). The MIC values ranged from 23.43 µg/mL observed in isolate S58, associated with the prevalent *S.* Typhimurium ST19 clone, to 312.50 µg/mL in *S.* Hadar ST33 (isolate S29), and up to 500 µg/mL in isolates S21, S34, S106, and S129, which are also linked to the *S.* Typhimurium ST19 clone.

The quantitative assessment of AgNPsol’s antibacterial activity against *Salmonella* spp. isolates revealed variations in the MIC values, indicating a diverse range of susceptibility across the isolates. Most strains exhibit relatively low MIC values, demonstrating increased sensitivity, with many values around or below 100 µg/mL. The highest antibacterial effectiveness was noted in one isolate (S58), part of the prevalent ST19 clone of *S.* Typhimurium (MIC = 23.43 µg/mL), followed by *S.* Hadar ST33 (136) and another *S.* Typhimurium ST19 isolate (S152) (MIC = 31.25 µg/mL). In contrast, the most resistant isolates included *S.* Typhimurium ST19 (S21, S34, S106, and S129 isolates) (MIC = 500 µg/mL) and *S.* Hadar ST33 (isolate S29) (MIC = 312.50 µg/mL) (Figure 8 and Supplementary Table S9).

#### 2.9.3. Anti-Adherence Activity of AgNPsol

**Figure 9:**
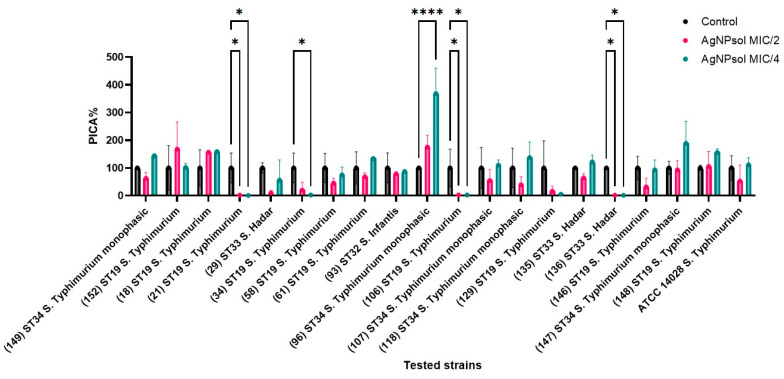
Graphical representation of the inhibitory effect on the adhesion capacity of *S. enterica* isolates treated with MIC/2 and MIC/4 of AgNPsol (Dunnett’s multiple comparisons test, n = 3 replicates, where * *p* < 0.05, and **** *p* < 0.0001).

The anti-adherence effect of AgNPsol on *S. enterica* isolates was evaluated at sub-inhibitory concentrations (MIC/2 and MIC/4), revealing variable impacts on *Salmonella* adherence capacity. Certain isolates exhibited a significant reduction in the adherence capacity, while others remained unaffected. Notably, *S.* Hadar ST33 (isolates S29 and S136); *S.* Infantis ST32 (isolate S93); and *S.* Typhimurium ST19 (isolates S21, S34, S58, S106, and S129) showed pronounced decreases in the adherence capacity at both MIC/2 and MIC/4, as evidenced by PICA values below the control. In contrast, some isolates, including *S.* Typhimurium ST19 (S18 and S148 isolates) and *S.* Typhimurium monophasic variant linked to the ST34 (isolate S96), highlighted high adherence percentages at these sub-inhibitory concentrations, indicating reduced susceptibility to AgNPsol’s anti-adherence effects (Figure 9).

#### 2.9.4. Extracellular Nitric Oxide Production

**Figure 10:**
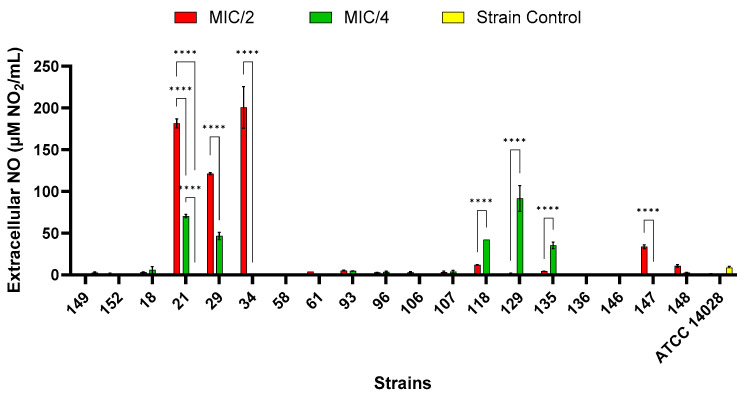
Extracellular NO content determined by the Griess reaction for AgNPsol in the presence of *S. enterica* strains (Sidak’s method, n = 3 replicates, **** *p* < 0.0001).

Exogenous nitric oxide (NO) at sub-lethal concentrations can cause bacteria in biofilms to transition from sessile states to planktonic, free-swimming states. In addition, it has been found that non-lethal levels of NO increase the susceptibility of certain biofilms to antimicrobial treatments [44]. As shown in Figure 10, the extracellular concentrations of NO increased with higher concentrations of AgNPsol in the S21, S29, S34, and S147 isolates, while for the S118, S129, and S135 isolates, the effect was reversed. For the isolates untreated with AgNPsol, the NO concentration is below the LOD (limit of detection) except for the S149 and S21 isolates and the reference strain (ATCC 14028). At MIC/2, strains belonging to different serovars and clones, including *S.* Typhimurium ST19 (S21), *S.* Hadar ST33 (S29), and *S.* Typhimurium monophasic variant linked to ST34 (S34) (*p* < 0.0001), had significantly higher levels of extracellular NO compared to the control isolate, as well as to the MIC/4 value. At MIC/4, the *S.* Typhimurium monophasic variant linked to ST34 (S118), *S.* Typhimurium ST19 (S129), and *S.* Hadar ST33 (S135) (*p* < 0.0001) had considerably higher levels of extracellular NO compared to the control isolate, as well as to the MIC/2 value (Figure 10). Different NO concentrations at MIC/2 and MIC/4 for strains indicate that cells use NO differently based on stress intensity. Some bacteria perceive AgNPsol as a moderate stressor, producing more NO as a stress signal or adaptation mechanism [45]. AgNPsol concentrations at MIC/4 may cause a peak reaction before inhibition or drive bacteria above the stress threshold, downregulating or degrading NO-producing pathways in strains belonging to the *S.* Typhimurium monophasic variant (S118), *S.* Typhimurium (S129), and *S.* Hadar (S135) [46].

#### 2.9.5. Advanced Oxidation Protein Products

**Figure 11:**
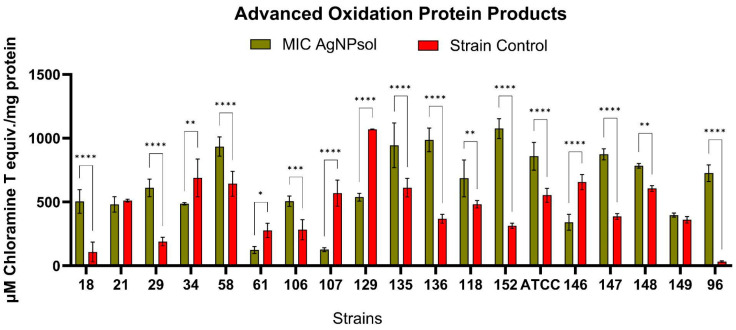
Oxidation protein products (AOPPs) in *Salmonella* spp. isolates treated with AgNPsol compared to the control isolates for 4 h incubation at 37 °C (Sidak’s method, n = 3 replicates, * *p* < 0.05, ** *p* < 0.01, *** *p* < 0.001, and **** *p* < 0.0001).

Protein oxidation was notably increased in most *Salmonella* spp. isolates treated with the AgNPsol MICs after 4 h of incubation. The highest levels of advanced oxidation protein products (AOPP) were detected in the 152 (1075.56 ± 78.11 µM Chloramine T equivalents/mg protein), S58 (934.96 ± 75.98), S135 (944.73 ± 175.09), and S136 (987.16 ± 92.32) isolates, also showing elevated AOPP values (Figure 11). These findings indicate that these isolates are particularly susceptible to the oxidative effects induced by AgNPsol, *S.* Typhimurium S152 isolate. displaying the highest susceptibility to protein oxidation among all the analysed isolates. Additionally, a significant increase in AOPP production compared to untreated control isolates was observed in the S18 (*p* < 0.0001), S29 (*p* < 0.0001), S106 (*p* < 0.001), S135 (*p* < 0.01), S118 (*p* < 0.01), ATCC (*p* < 0.0001), S147 (*p* < 0.0001), S148 (*p* < 0.01), and S96 (*p* < 0.0001) isolates. These results suggest that AgNPsol effectively induces oxidative stress in bacterial cells, leading to increased protein oxidation. This increase underscores the potential of AgNPs as antimicrobial agents, as oxidative stress can compromise bacterial viability and disrupt essential cellular functions [34,47]. AgNPs are known to generate ROS upon contact with bacterial cells, which leads to the oxidation of cellular components, including proteins [48,49]. These findings indicate that AgNP treatment effectively induces oxidative stress in specific *Salmonella* isolates, adversely affecting the protein structure and function and further supporting the antimicrobial potential of AgNPs in targeting bacterial pathogens.

In some cases, a significant reduction in microbial protein oxidation was observed, especially in the case of the S61, S107, S129, and S146 isolates, which could indicate a higher resistance to the oxidative effects of AgNPsol. This paradoxical effect suggests inter-strain variability, which can be explained by genetic or metabolic differences between isolates. The reduction of AOPP may indicate either a natural protection against protein oxidation or an inhibition of ROS produced by AgNPs under certain conditions. Some isolates may express mechanisms of resistance to oxidative stress, such as antioxidant enzymes (superoxide dismutase, catalase) or efflux mechanisms to eliminate AgNPs from the cell [50]. At AgNP concentrations below 62.5 µg/mL, cells experience oxidative stress without immediate lysis, leading to an accumulation of ROS and oxidised proteins in the extracellular environment. This sub-lethal stress allows cells to remain metabolically active, which promotes the production and release of oxidative by-products, resulting in higher extracellular AOPP levels [51,52,53]. In contrast, at higher concentrations, AgNPs may cause rapid cell lysis, reducing the opportunity for cells to produce AOPP over time.

### 2.10. Cell Viability

The HEK-293 cell line, derived from human embryonic kidney cells, is a suitable in vitro model for studying the toxic effects of nanoparticles on human renal tissue [54], given the kidneys’ critical role in eliminating metals and nanoparticles from the body [55]. Following administration, AgNPs can accumulate in the kidneys, where they may induce oxidative stress, inflammation, and cell death [56,57].

**Figure 12:**
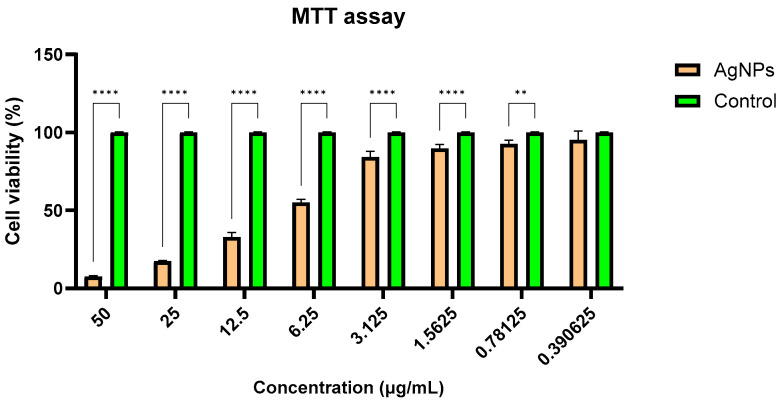
Cytotoxicity assessment of AgNPs through MTT test after 24 h. The assay was performed in triplicate, and the differences between the control and AgNPs were considered significant for *p* < 0.05 (** *p* < 0.01 and **** *p* < 0.0001).

The results demonstrate a dose-dependent cytotoxic effect on HEK-293 cells (Figure 12), the IC50 value being calculated at 7.84 ± 0.52 µg/mL. As the AgNPs concentration decreases, the cell viability increases, with almost no cytotoxicity for doses below 1 µg/mL. These results are in accordance with previous literature on AgNPs, which showed even a lower IC50 on HEK-293 cells, 12.135 ng reported by Liu et al. in 2021 [58] and 1.522 µg/mL established by Kaur at al. in 2024 [59].

## 3. Discussion

One of the most important public health concerns is represented by the enteric foodborne pathogenic bacteria to which *Salmonella* belongs. There have been described and classified more than 2600 *S. enterica* serovars [60]. The present study has elucidated the AR profiles and genotypic markers, soluble virulence factors, and biofilm production ability of *Salmonella* spp. isolated from Romanian outpatients’ stool samples at Synevo Central Laboratory Bucharest, South Romania, in 2019, the VMs associated with resistant clones and alternative solution to conventional antibiotics based on AgNPsol to fight against the resistant and virulent clones. We demonstrated a low incidence of ESBL-encoding genes in 201 *Salmonella* spp. strains isolated from outpatients, of which 65.05% were obtained from children under 4 years old. The detection of ESBL-producing isolates is alarming due to their resistance to cephalosporins, the preferred antibiotics for treating salmonellosis in children. This resistance complicates treatment strategies, leading to limited therapeutic options and potentially severe clinical outcomes. The presence of ESBL encoding genes like *bla*_TEM_, *bla*_SHV_, and *bla*_CTX-M_, even at relatively low frequencies, is critical, because it not only impacts individual treatment success but also poses a risk of horizontal gene transfer, potentially amplifying resistance across different bacterial populations [61]. The identification of PMQR genes such as *qnrB* and *qnrA* in ciprofloxacin-susceptible isolates is particularly troubling. These genes facilitate the development of high-level fluoroquinolone resistance, an issue compounded by their ability to promote chromosomal mutations associated with increased resistance. This scenario suggests that *Salmonella* strains initially susceptible to fluoroquinolones could evolve rapidly towards resistance, complicating the management of outbreaks and routine infections [62,63]. The presence of these genes necessitates multifaced public health responses, including enhanced surveillance, strict infection control practices, and the judicious use of antibiotics. Additionally, it highlights the need for ongoing research into alternative treatments, such as the use of AgNPs, to combat antibiotic-resistant bacteria.

To estimate the virulence potential of *Salmonella* strains soluble VF and four of the most encountered VMs in *S. enterica* represented by *invA*, *spvC*, *pldA*, and held genes were investigated. The virulence potential includes the presence of pore-forming toxins (PFTs) [hemolysins β and α (88% of the strains), lecithinase (13%), lipase (49%)], proteases [caseinase (29%), and gelatinase (83%)]; amylase (17%); and aesculin hydrolysis (89%). Among the key VFs, PFTs with cytotoxic properties, notably α and β hemolysins, play a significant role in lysing and damaging eukaryotic cell membranes to facilitate nutrient acquisition, particularly iron. These toxins are also capable of activating cell signalling pathways that promote cytoskeletal reorganisation and stimulate various defence and innate immune responses, critical activities for the bacteria’s survival and proliferation within the host [64]. Both haemolytic phenotypes are considered as VMs with clinical significance contributing directly or indirectly to bacterial invasion and dissemination [65,66]. The third most frequently identified VF in *Salmonella* strains was gelatinase, a proteolytic enzyme that degrades gelatine into amino acids, peptides, and polypeptides. This enzyme plays a role in host cell invasion, crossing the cell membrane to facilitate cellular migration into host tissues [67]. Gelatinase has been demonstrated to play an essential role in biofilm formation via the quorum-sensing system by modulating signalling pathways [68] and aiding bacterial translocation across intestinal cell layers [69], which is significant for persistent infection and resistance to treatment.

One of the most studied VF considered a biomarker for the *Salmonella* genus is represented by invasion A (*invA*)—an outer membrane factor responsible for initiating the infection [70] revealed by the majority of the analysed strains (98.63%). The *spv* operon (Salmonella plasmid virulence) is especially significant in *Salmonella* spp. This operon includes five genes: *spvR*, a positive regulator, and four structural genes: *spvA*, *spvB*, *spvC*, and *spvD* [71]. There have been observed positive associations between the encoding genes for different antibiotic classes and VMs: *bla*_SHV_/*spvC*, *qnrB/spvC*, and *sul1/spvC* (*p* < 0.05) and *tet(A)/spvC* and *bla*_TEM_/*spvC* (*p* < 0.001). Of great concern is the presence of the *spvC* virulence gene in 6.85% of the isolates, which is consistent with other studies from Egypt and Spain [72,73]. The *spvC* gene aids in the systemic dissemination of *Salmonella* strains, underscoring the potentially high virulence of these strains [74]. The observed associations between genes encoding for different antibiotic classes and VMs such as *bla*_SHV_, *qnrB*, and *sul1* with *spvC* underline the dual challenge of managing infections caused by these isolates due to their virulence coupled with AR. This complicates treatment strategies, as the bacteria are not only more capable of causing severe infection but are also resistant to conventional treatments. Understanding these factors is critical for developing more effective therapeutic and preventive measures against salmonellosis.

WGS of 19 *S. enterica* isolates, obtained from children aged 2 months to 6 years, revealed the presence of four serovars: *S.* Typhimurium and its monophasic variant, outbreak strains responsible for human infections transmitted through the food chain and, in the second and third place on salmonellosis after EFSA, 2021, *S. enterica* Hadar, widespread in the USA and Europe (Spain, Italy, Greece, and Switzerland, including Hungary, a neighbouring country of Romania), and *S. enterica* Infantis (reported also in the same year in Spain, Slovakia, Slovenia, Poland, the Netherlands, Italy, Hungary, Croatia, Belgium, and Austria) [75,76,77,78,79,80,81]. These serovars were further associated with specific STs, indicating the presence of circulating clones. Notably, S. Typhimurium was linked to the widespread sequence types ST19; *S.* 4,[5],12:i:- to ST34, *S. enterica* Hadar was associated with ST33, and *S. enterica* Infantis was linked to ST32. This suggests the ongoing circulation of these established clonal lineages within the paediatric population.

Therefore, 93% of *S.* Typhimurium circulating in ambulatory children from Bucharest population were TEM-1 producers disseminated by IncB/O/K/Z_2, IncFIB(S)_1, IncFII(S)_1, IncR_1, IncX3_1, IncX1_1, IncFIB(AP001918)_1, ColRNAI_1, Col440I_1, and Col(VCM04)_1 plasmid and belong to ST34 and ST19 clones. *S.* Typhimurium and its monophasic variant *S.* 4,[5],12:i:- represents major public health issues in Europe, particularly due to their association with two major clones: ST19 and ST34 implicated in numerous foodborne outbreaks, with ST34 being the most frequently detected in recent years in multiple European countries, including the United Kingdom, Denmark, Portugal, France, Germany, and Italy [82]. Its widespread presence in food production systems, especially broilers, and its transmission through the food chain to humans has made it a key focus for food safety and public health efforts.

It was noticed that *S.* Hadar linked to ST33 was associated with tetracycline resistance (75% of the isolates), very similar to *S.* Hadar isolated from stool samples between 2005 and 2010 in Switzerland [79].

Using WGS, we demonstrated that a MDR *S. enterica* isolate from a one-year-old child belonged to the ST32 sequence type and carried a combination of resistance genes, including *sul2*, *aadA5*, and *dfrA17*. These genes were disseminated via IncI_1_Alpha plasmids, contributing to its MDR profile. Franco et al. (2015) [83] provided the first evidence of an ESBL-producing *S.* Infantis ST32 clone in Italy. This clone, along with a single-locus variant, harboured a conjugative pESI-like megaplasmid containing the *bla*_CTX-M-1_ gene, which contributes to its AR profile. Originally disseminating from Israel in 2008, the clone became prevalent in the Italian broiler chicken industry and spread to humans through the food chain. Hindermann et al. (2017) [84] identified the presence of the *S.* Infantis ST32 clone in food and human isolates collected in Switzerland between 2010 and 2015 and previously identified in Hungary, Poland, Austria, Germany, Israel, and Japan [81,85]. The study also highlighted the emergence of a MDR *S.* Infantis lineage harbours the *bla*_CTX-M-65_ gene, which confers ESBL production, further complicating treatment options. Papić et al. recently reported that the MDR, pESI-positive *S.* Infantis clone has become the dominant strain in Slovenian broilers and humans over the past decade [86].

Furthermore, it was observed that circulating *S. enterica* clones revealed the presence of different ARG configurations, which could potentially contribute to their multidrug resistance profiles, for, e.g., in ST19 (4): *mph(A)*-*dfrA5*-*sul1*; *bla*_TEM-1_-*dfrA5*-*sul1*-*qnrA1*-*cmlA5*; *bla*_TEM-1_; *bla*_TEM-1-_*dfrA12*-*aadA2*-*cmlA1*-*aadA1*-*sul3*; in ST34 (3): *bla*_TEM-1_-*tet(B)*-*aph(6)*-*Id*-*aph(3″)*-*Ib*-*sul2*; *bla*_TEM-1_-*tet(B)-aph(6)-Id*-*aph(3″)-Ib*- *ant(2″)-Ia-sul2*; *bla*_TEM-1_-*dfrA5*-*sul1*-*qnrA1*-*cmlA5*; and in the last two clone ST33 (1): *tet(A)*-*aph(6)-Id*-*aph(3″)-Ib*-*qnrB19;* and ST32 (1): *dfrA17*-*aadA5*-*sul2*. ST19 was disseminated by IncB/O/K/Z_2; IncFIB(S)_1; IncFII(S)_1; IncX3_1; IncX1_1; ColRNAI_1; Col440I_1; ST34 by Col (VCM04) _1; ST33 by Col8282_1; Col440I_1; Col (VCM04)_1; and ST32 by IncI1_1_Alpha plasmid replicons.

The identified clones were associated with VFs, which encode fimbriae (*fimI*, *fimC*, *fimD*, *fimH*, *fimF*, *lpfE*, *lpfD*, *lpfC*, *lpfB*, and *lpfA* genes); flagella (*csgC*, *csgA*, *csgB*, *csgD*, *csgE*, *csgF*, and *csgG* genes); outer membrane proteins (*sinH* and *ompA* genes); Type III Secretion System (T3SS), specifically encoded by *Salmonella* Pathogenicity Island 1 (SPI-1): *invH*, *invF*, *invG*, *invE*, *invA*, *invB*, *invC*, *invI*, *invJ*, *sicA*, *sipB/sspB*, *sipC/sspC*, *sipD*, *sipA/sspA*, and *sicP* genes; SPI-1 and SPI-2: *sopD* gene; SPI-2: *SsaU*, *SsaT*, *SsaS*, *SsaR*, *SsaQ*, *SsaP*, *SsaO*, *SsaN*, *SsaV*, *SsaM*, *SsaL*, *SsaK*, *SsaJ*, *SsaI*, *SsaH*, *SsaG*, and *spiC* genes; SPI-2-encoded effectors: *sseG*, *sseF*, *sscB*, *sseE*, *sseD*, *sseC*, *sscA*, *sseB*, and *sseA* genes; virulence plasmids: *spvC*, *B*, and *R* genes; enterobactin: *entB*, *entA*, *fepC*, and *fepG* genes; superoxide dismutase: *sodCI* gene; proteins that contributes to the bacteria’s ability to evade the host immune system: *mig-14* gene; resistant to complement killing proteins: *Rck* gene (with the exception of the ST34, ST33, and ST32 clones); and other effectors and regulators: *avrA*, *sseL*, *sseK2*, *sseI/srfH*, *slrP*, *sseK1*, *sopD2*, *sopA*, *sopE2*, *steC*, *sseJ*, *steB*, *sifB*, *steA*, *gogB*, *spaO*, *spaP*, *spaQ*, *spaR*, *spaS*, *sopB/sigD*, *orgA*, *orgB*, *orgC*, *pipB*, *pipB2*, *prgH*, *prgI*, *prgJ*, *prgK*, *sptP*, *sifA*, *sifA*, *pipB2*, *mgtB*, and *mgtC* genes.

In Romania, there are very few studies available regarding the presence of MDR *Salmonella* strains belonging to different serovars in various isolation sources. For example, Tîrziu et al. [87] in 2015 reported a high rate of MDR *Salmonella* contamination in raw chicken meat, alongside the detection of eight circulating serotypes of *S. enterica* subsp. *enterica* in Constanța, a county in Romania located on the western shore of the Black Sea. This poses a significant public health risk due to the potential for these resistant strains to cause severe infections in humans. Mihaiu et al. [88] demonstrated that chicken and pork meat can act as significant sources of human exposure to MDR *Salmonella*, posing a risk for the transmission of resistant foodborne diseases. The study identified thirteen *Salmonella* serovars, with Infantis and Typhimurium being the most prevalent, highlighting the potential public health threat posed by these contaminated meat products. Between January 2016 and April 2020, a study published in Romania provided an overview of the genetic diversity of the *S.* Enteritidis strains involved in human infections. The study identified the occurrence of the sequence type ST11, a globally prevalent clone associated with significant public health concerns. This clone was detected in patients from various counties in Romania and was linked to strains reported in major international outbreaks during the same period [89].

In addition to *S.* Enteritidis, *S.* Typhimurium and its monophasic variant are among the most frequently reported serovars in human cases across Europe, including Romania. These isolates are primarily associated with foodborne outbreaks and are linked to contaminated poultry and pork products [90,91]. Moreover, *S.* Infantis, a serovar increasingly recognized for its MDR, has been reported in Romania, as well as other European countries [92].

Given that the *S. enterica* isolates analysed by WGS exhibited ARGs for one to five antibiotic classes, we investigated an alternative to conventional antibiotics based on AgNPs synthesised through a solvothermal method, which could offer a potential solution to combat MDR isolates. In qualitative screening, AgNPsol exhibited efficacy against 50% of *S. enterica* isolates, showing selective effectiveness across different serovars. The highest activity was observed against *S.* Infantis, while reduced efficacy was noted against certain other serovars, such as *S.* Typhimurium ST19. Quantitative MIC assessments revealed substantial variability in susceptibility, with the most susceptible isolate identified as part of the *S.* Typhimurium ST19 clone. In contrast, the most resistant isolates included additional *S.* Typhimurium ST19 and *S.* Hadar ST33, indicating isolate-specific differences in resistance to AgNPsol. Additionally, *Salmonella* isolates varied in their adherence inhibition capacities to AgNPsol, suggesting that AgNPsol could serve as an alternative therapeutic for *S. enterica* infections in paediatric patients, particularly those aged 2 months to 6 years, where it demonstrated a consistent antibacterial efficiency.

The antibacterial mechanism of AgNPs against *Salmonella* primarily involves disruption of the bacterial inner membrane, resulting in membrane dysfunction. Seong and Lee (2017) [93] demonstrated that AgNPs increased the permeability of the inner membrane without affecting the outer membrane. Additionally, AgNPs induced the accumulation of ROS and intracellular Ca^2+^; however, ROS were not directly implicated in the bactericidal effects. Instead, the key factor contributing to the antibacterial activity of AgNPs was the disruption of the inner membrane, which led to bacterial cell death [84].

Furthermore, AgNPs are known to produce ROS, which may cause bacteria to upregulate nitric oxide synthase (NOS) or other NO-producing enzyme systems in a protective or signalling response [94]. NO produced under oxidative stress can serve a variety of functions, including protection against ROS, signalling the shift from biofilm (sessile) to planktonic stages, and facilitating biofilm dispersion, which may render the bacteria more vulnerable to antimicrobial treatments [95]. In strains where NO declines as AgNP concentrations rise, excessive oxidative stress may exceed the cells’ ability to create NO or cause greater breakdown or transformation of NO into less reactive molecules [50,96]. At high AgNP concentrations, bacteria may use particular enzymes, such as nitric oxide dioxygenase or nitrite reductase, to quickly metabolise or detoxify NO, limiting its buildup [97]. Alternatively, at greater AgNP concentrations, cell damage may completely limit NO production pathways, resulting in a decline in NO levels when cells die or lose metabolic activity [98].

The results of the AOPP assays suggest that AgNPs effectively inhibit certain strains of *Salmonella* by inducing oxidative stress, although not all strains respond uniformly. These findings could aid in identifying *Salmonella* strains that are particularly susceptible to AgNPs, indicating their potential as selective antimicrobial treatment. However, the variability in oxidative responses indicates the need for additional studies to identify the mechanisms of resistance to AgNPs and to optimise their use as a targeted antimicrobial agent. This response variability is consistent with previous studies showing that AgNPs’ antimicrobial properties involve multiple mechanisms, including ROS generation, membrane disruption, and interference with protein function. These mechanisms influence bacterial growth and stress response pathways differently depending on the strain’s resilience and ROS-scavenging abilities [99].

The antimicrobial efficacy of AgNPs against *Salmonella* isolates from various sources is well documented. For instance, AgNPs have demonstrated potent antimicrobial and antibiofilm properties, effectively disrupting and eliminating biofilms produced by *Salmonella* Enteritidis isolated from poultry environments [100]. In another study, AgNPs significantly reduced *S.* Typhimurium infection in rats, as evidenced by a substantial decrease in microbial load in faeces within 5–6 days of treatment [101]. Similarly, Sanguiñedo et al. 2018 [102] reported that biogenic AgNPs exhibit strong bacteriostatic and bactericidal properties against MDR *S.* Typhimurium isolates. Chiao et al. (2012) [103] further highlighted the enhanced antibacterial effects of nanohybrids combining nanoscale silicate platelets (NSPs) with AgNPs, which significantly reduced *Salmonella*-induced septicaemia and mortality in chicks. These nanohybrids also minimised silver accumulation in tissues, suggesting that NSP enhances safety by reducing tissue deposition. offering a promising and safer option for treating enteric bacterial infections [94].

Additionally, Yakoup et al. (2024) [104] explored the biofilm matrix as a scaffold for synthesizing of AgNPs to combat MDR bacteria. These biofilm-synthesised AgNPs exhibited significant antibacterial activity against a broad range of bacterial strains, including *Salmonella enterica*, at low concentrations. When combined with phage ZCSE9, these biofilm AgNPs exhibited synergistic antibacterial effects, effectively lowering the MIC values and reducing toxicity against *S. enterica*. This combination enhances their potential as a safer and more effective antimicrobial treatment.

The obtained IC50 value on HEK-293 cells indicates moderate toxicity of the AgNPs tested, as higher concentrations of AgNPs significantly reduced cell viability, while doses below 1 µg/mL exhibited minimal or negligible cytotoxic effects, preserving cell viability. Factors such as particle size, surface modifications, and aggregation state can significantly influence the biological responses of HEK-293 cells to AgNPs, explaining the differences between different reports. Furthermore, encapsulation can reduce the direct contact between AgNPs and HEK-293 cells and can ensure a slow release of silver ions (Ag^+^) (e.g., polydopamine, starch, and fungal chitosan) [105,106,107]. This slow release provides prolonged antimicrobial activity without inducing a toxic peak at the beginning of exposure [108]. Surface modifications can prevent direct interaction with cell membranes, reducing toxicity without affecting antimicrobial activity [109,110,111]. The exposure time depends on how long AgNPs remain in the body, which is influenced by the size and shape of the nanoparticles, the elimination system, and the chemical modifications [112]. The kidneys filter AgNPs from the blood, and then, they can be eliminated in urine within a few days or weeks, depending on their size and stability [113]. The tested AgNPs are spherical with a size of 33 nm according to our previews publish data [114]; it is generally known that NPs smaller than 10 nm can more easily penetrate cell membranes, and consequently, the cytotoxicity is higher [115,116].

## 4. Materials and Methods

### 4.1. Sample Collection and Antibiotic Susceptibility Testing

Faecal samples were collected from outpatients at the Synevo Central Laboratory in Bucharest, Romania, in 2019. Appropriate measures were taken to ensure patient privacy and maintain the confidentiality of their information. The isolated bacterial strains were identified as *Salmonella* spp. using MALDI-TOF MS MBT Smart, with MSP 96 target polished steel BC (Bruker system, Berlin, Germany). The susceptibility to different antibiotic classes (Table 5) of each strain was tested using WalkAway 96 Si MicroScan system (Siemens Healthineers, Erlangen, Germany). Antibiotic resistance profiles were assigned according to the Clinical and Laboratory Standards Institute (CLSI, 2020) [117] performance standards belonging to the *Enterobacterales* order.

### 4.2. Evaluation of the Soluble Enzymatic Virulence Factors

An overnight culture of *Salmonella* strains was tested for several VFs, including pore-forming toxins (hemolysins, lecithinase, and lipase); proteases (caseinase and gelatinase), amylase; and aesculin hydrolysis. The VF production after incubation at 37 °C, 24 h was evaluated on specific culture media: hemolysin: cultures on 5% sheep blood agar; colourless zone indicates haemolysis; lipase: cultures on 1% Tween 80 agar; a precipitation zone shows positive reaction; lecithinase: cultures on 2.5% yolk agar; a clear zone indicates activity; caseinase and gelatinase activity: caseinase on 15% casein agar and gelatinase on 3% gelatine; positive results shown by white or colourless zones, respectively; amylase detection: cultures on 1% starch agar; Lugol’s solution reveals hydrolysis with a yellow ring around colonies; aesculin hydrolysis: cultures on Fe^3+^ citrate medium; a black precipitate signals positive activity due to esculetol release [118,119].

### 4.3. Biofilm Formation Assay

Bacterial cultures grown in Tryptic Soy Broth (TSB) (Oxoid, Hampshire, UK) supplemented with 2.5% glucose in 96-well plates were rinsed with phosphate-buffered saline (PBS), and cells were fixed with methanol for 5 min and stained with 1% crystal violet for 15 min. Following three PBS washes, cells were re-suspended in 33% acetic acid, and absorbance at 490 nm was measured using a Thermo Scientific Multiskan FC spectrophotometer. Each sample was tested in triplicate.

For assessing biofilm formation levels, the cut-off OD (ODc) was defined as three standard deviations above the mean OD of the negative controls, following Stepanović et al. (2007) [120]. The average OD and standard deviation were then calculated for each set of triplicates. The ODc value was subtracted from each mean OD, and results with negative values, i.e., OD ≤ ODc, were discarded, as these do not meet the cut-off value for biofilm formation. Strains for which ODc < OD ≤ 2 ×ODc were categorised as having a weak capacity for biofilm formation. Moderate biofilm formation capacity was assigned to strains for which 2 ×ODc < OD ≤ 4ODc. No strong biofilm producers, i.e., OD > 4 ×ODc, were revealed.

### 4.4. Molecular Characterization of Salmonella Strains

#### 4.4.1. DNA Extraction

Microbial DNA was obtained from 201 *Salmonella* strains that shows resistance in different levels to at least one tested antibiotic using an adapted alkaline extraction method, as previously described [121]. Briefly, bacterial colonies 1–5 were suspended in 20 µL solution of 0.05 M NaOH and 0.25% SDS, then heated at 95 °C for 15 min. After cooling, 180 µL of 1 × TE buffer was added, and the suspension was centrifuged at 13,000 rpm for 3 min. The supernatant was kept at 4 °C and used in PCR reactions.

#### 4.4.2. Polymerase Chain Reaction for ARGs and VFs Detection

PCR amplification was performed for each isolated strain exhibiting resistance to at least one tested antibiotic using specific primers for the most frequent antibiotic resistance and virulence-encoding genes (Supplementary Table S1A,B). The PCR tubes were prepared using 8 μL reaction buffer, 10 μL Green Master Mix (ThermoScientific, Waltham, MA, USA), 1 μL DNA, and 0.5 μL of forward and reverse primers [122,123,124,125,126,127,128,129]. The resulting amplicons were visualised using agarose (1%) gel electrophoresis and transilluminator.

#### 4.4.3. Whole-Genome Sequencing (WGS) and Bioinformatics Analyses of *Salmonella* Isolates

We conducted WGS and bioinformatics analysis on a subset of *Salmonella* isolates from stool samples collected at the Synevo Central Reference Laboratory in Bucharest, Romania. Out of a total of 309 isolates, 19 isolates were selected for WGS based on their AR profiles. Genomic DNA was extracted using the DNeasy UltraClean Microbial Kit (Qiagen, Hilden, Germany). Library preparation was performed with the Nextera DNA Prep Library Kit (Illumina, San Diego, CA, USA), and sequencing was carried out using Illumina’s MiSeq platform (V3, 600 cycles), allowing for high-throughput and detailed genomic analysis. De novo assembly of raw reads was performed using the Shovill v1.1.0 pipeline [130]. Annotation was conducted with BAKTA v1.9.4 [131], alongside ABRicate v1.0.0 [132], which utilised the NCBI and VFDB [133] databases to establish the ARGs and VF profiles. MGE predictions have been done using a workflow similar to our previous ones [134]. Sequence types of the isolates were assigned through the Multilocus Sequence Typing (MLST) tool [135], following the Achtman scheme. The *Salmonella* In Silico Typing Resource (SISTR) v1.1.2 [136] command-line tool and Pathogenwatch [137] were used to determine the serotypes of the *Salmonella* isolates. The resulting contig sequences were further employed for pangenome analysis with Roary [138], based on the annotations obtained with BAKTA. The pangenome tree has been visualised using the Phandango [139] online tool. Additionally, an in-house script was used to identify gene clusters in the contig sequences. Notably, one isolate (S146) could not be annotated and is absent from the pangenome tree image.

#### 4.4.4. Conjugation Assays Were Performed on Seven Representative Isolates

Characterised by WGS and demonstrating tetracycline resistance (MIC > 8 µg/mL), using *E. coli* J53 (sodium azide-resistant) as the recipient strain. Transconjugants were selected on MacConkey agar (Oxoid, Hampshire, UK) containing sodium azide (150 mg/L) and tetracycline (8 mg/L), followed by determination of Cf. Subsequent PCR screening of transconjugants was conducted to confirm the conjugative transfer of tetracycline resistance genes.

$$Cf=\frac{\mathrm{colonies}\;\mathrm{number}}{3}\times \mathrm{dilution}\;\mathrm{factor}$$

#### 4.4.5. GenBank Accession Numbers

The raw whole-genome sequencing data for the analysed samples were deposited in the GenBank Sequence Read Archive (SRA) under the accession code PRJNA1185014 (https://www.ncbi.nlm.nih.gov/bioproject/PRJNA1185014), URL (accessed on 12 November 2024).

### 4.5. Antimicrobial, Anti-Biofilm Activity, and the Impact on Biochemical Processes of AgNP Solutions (AgNPs)

An alternative solution to conventional antibiotics based on AgNPs [synthetized by the classical and hydrothermal methods and characterised using the FTIR, SEM, TEM, DLS, and XRD methods as previously described [114]] was tested by qualitative and quantitative methods against 19 isolates of *S. enterica* investigated at the genotypic level through WGS, along with *S. enterica* ATCC 14028, to provide a comprehensive evaluation of the NPs antimicrobial potential.

#### 4.5.1. Qualitative Screening of AgNP Against Selected *S. enterica* Strains

A modified diffusion method was utilised to screen the antimicrobial effects of AgNPs (encoded Ag2NP and Ag3NPsol), for a total of 19 *S. enterica* strains. Microbial suspensions (0.5 McFarland) obtained from 24-h cultures in sterile distilled water were uniformly applied to Mueller–Hinton agar (MH) media (Oxoid, Hampshire, UK). Next, 10 μL of 0.01 mg/mL Ag2NPs and 1 mg/mL AgNP solution were applied to the inoculated media, followed by incubation (37 °C for 24 h), measurement of the diameters of growth inhibition zones, and their conversion into arbitrary units using a scale where a value of 0 indicated no inhibition (arbitrary unit, AU = 0), a value of 1 corresponded to an inhibition zone diameter up to 10 mm (AU = 1), and a value of 2 represented an inhibition zone diameter of 11–20 mm (AU = 2) [121,140].

#### 4.5.2. Quantitative Evaluation of AgNPsol Efficacy

The AgNPsol was evaluated quantitatively using a broth microdilution method in Mueller–Hinton broth in order to determine the MIC. For this, AgNPsol were serially diluted two-fold across concentrations ranging from 500 to 0.97 µg/mL in 100 μL of broth media. Each well from 1 to 10 of the dilution series was then inoculated with 20 μL of a 0.5 McFarland bacterial suspension derived from bacteria cultured for 24 h at 37 °C on Plate Count Agar media (Oxoid, Hampshire, UK). Growth monitoring was conducted using a Thermo Scientific Multiskan FC multi-reader (Waltham, MA, USA) by measuring the optical density at 600 nm. The assay included controls: well no. 11 contained untreated cultures serving as the positive control, and well no. 12 served as the sterility (negative) control.

#### 4.5.3. The Influence of AgNPs on Adherence Capacity

The adherence inhibition assay for the *S. enterica* strains was conducted similarly to the biofilm formation assay, using sub-inhibitory concentrations of AgNPsol to assess the impact on microbial adherence. The percentage of adherence inhibition (PICA%) was calculated based on absorbance values and the previously described relationship [140].

#### 4.5.4. Extracellular Nitric Oxide Quantification

The nitric oxide (NO) levels were measured using a spectrophotometric assay based on the Griess reagent, following established methods with slight adjustments [49]. The resulting supernatant was combined with 2% sulphanilamide solution in 5% H_3_PO_4_ and 0.1% solution of N-(1-naphthyl)-ethylenediamine in water in a volume ratio 6:5:5 (*v*:*v*:*v*). After allowing 30 min for the azo dye to form, the absorbance was read at 540 nm. A standard curve was prepared with NaNO_2_ at concentrations between 1 and 100 μM (R^2^ = 0.9998) for NO quantification.

#### 4.5.5. Advanced Oxidation Protein Products Quantification

The concentration of microbial advanced oxidation protein products (AOPPs) was done using a spectrophotometric assay adapted from Quinteros et al. (2016) [49]. A 1 McFarland microbial suspension was prepared in PBS from a 24-h culture, and the MIC values specific to each strain of AgNPs were considered. Each sample containing 500 μL AgNP solution in TSB and 100 μL microbial suspension was incubated for 4 h at 37 °C, then centrifuged at 10,000 rpm for 10 min. Following centrifugation, 0.1 mL of the supernatant was combined with 50 μL potassium iodide (1.16 M) and 50 μL acetic acid. The absorbance was read at 340 nm. Controls consisted of TSB, KI, and acetic acid, while blanks included TSB with AgNPs and PBS without microbes. The AOPP concentration was calculated using a chloramine T calibration curve (10–100 μM, R^2^ = 0.9963) and expressed as μmol chloramine T equivalent per mg protein, based on protein quantification by the Bradford assay [141].

### 4.6. Biocompatibility

Human embryonic kidney cells (HEK-293 cell line; catalogue number ATCC CRL-1573, Manassas, VA, USA) were seeded at a density of 3 × 10^4^ cells/well in 96-well plates containing Eagle’s Minimum Essential Medium supplemented with 10% foetal bovine serum and cultured overnight at 37 °C in a humidified atmosphere with 5% CO_2_. Following adhesion, the cells were exposed to AgNPs for 24 h. Cytotoxicity was assessed with the MTT assay (Sigma-Aldrich, St. Louis, MO, USA), the resulting purple formazan crystals, produced by viable cells, being dissolved in 2-propanol and absorbance measured at 595 nm on a FlexStation 3 microplate reader (Molecular Devices, LLC, San Jose, CA, USA).

### 4.7. Statistical Data Analysis

Data on the antimicrobial, anti-adherence activities, NO content, and AOPP of AgNPsol were analysed through 3 replicates using GraphPad Prism v10 (GraphPad Software, San Diego, CA, USA), with the results expressed as means ± SD. All analyses were assessed using a two-way ANOVA, followed by Dunnett’s multiple comparisons test to adjust for multiple comparisons. Statistical significance was set at *p* < 0.05.

## 5. Conclusions

This study highlights a significant incidence of antibiotic-resistant *Salmonella* strains in Romanian patients, with prevalent ARGs such as *bla*_TEM_, *tetA*, and *sul1* alongside virulence factors like *invA* and *spvC*, suggesting a potential co-selection of resistance and virulence. The efficacy of AgNPs against certain MDR *Salmonella* strains presents a promising alternative, especially for paediatric cases, although serotype-specific variability highlights the necessity of tailored approaches. These findings emphasise the urgent need for continuous surveillance, novel antimicrobial strategies, and stringent food safety measures to limit the spread and public health impact of virulent and resistant *Salmonella* clones.

## Figures and Tables

**Table 1:** The VFs produced by *Salmonella* strains by age group.

Bacterial Strain	Age Group	Number of Strains (%)	Isolation Source	β- and α-Haemolysins (%)	Lecitinase (%)	Amylase (%)	Lipase (%)	Caseinase (%)	Gelatinase (%)	Esculin Hydrolysis (%)
*Salmonella* spp.	0–4	201 (65.05%)	Stool samples	201 (100%, β)	38 (19%)	45 (22%)	108 (54%)	80 (40%)	189 (94%)	201 (100%)
	5–14	41 (13.27%)		41 (100%, β)	2 (5%)	7 (17%)	33 (80%)	5 (12%)	37 (90%)	41 (100%)
	15–24	7 (2.27%)		5 (71%, α)	0	1 (14%)	5 (71%)	5 (71%)	2 (29%)	5 (71%)
	25–44	33 (10.68%)		15 (45%, α)	1 (3%)	0	2 (6%)	0	12 (36%)	21 (64%)
	45–64	12 (3.88%)		8 (66%, α)	0	0	2 (17%)	0	11 (91%)	4 (33%)
	>65	15 (4.85%)		3 (20%, α)	0	0	1 (7%)	0	5 (33%)	3 (20%)

**Table 2:** ARGs in *S. enterica* isolated from outpatients. Different colors were used to highlight the affiliation of strains carrying different antibiotic resistance genes to different clones: blue for ST34; red for ST19; pink for ST33; and purple for ST32.

Antibiotic Classes	*ARGs*	0–4 Years (%)	Total No of Strains-17	6 Years (%)	Total No of Strains-2	STs			
						34	19	33	32
β-lactams	*bla*_TEM-1_	70.58	12	100	2				
Aminoglycosides	*aadA1*	0		50	1				
	*aadA2*	0		5.88	1				
	*aadA5*	5.88	1	0					
	*ant(2* *″* *)-Ia*	0		50	1				
	*aph(3* *″* *)-Ib*	35.29	6	50	1				
	*aph(6)-Id*	35.29	6	50	1				
Quinolones	*qnrA1*	35.29	6	50	1				
	*qnrB19*	17.64	3	0					
Sulphonamides	*sul1*	47.05	8	0					
	*sul2*	23.52	4	50	1				
	*sul3*	0		50	1				
Tetracyclines	*tet(B)*	11.76	2	50	1				
	*tet(A)*	17.64	3	0					
Macrolides	*mph(A)*	11.76	2	0					
Thrimethoprim	*dfrA5*	47.05	8	0					
	*dfrA12*	0		50	1				
	*drfA17*	5.88	1	0					
Chloramphenicol	*cmlA1*	0		50	1				
	*cmlA5*	35.29	6	0					

**Table 3:** Distribution of serovars among *Salmonella* isolates recovered from human stool samples.

Serotype	Antigen Structure	Number of Isolates	ST
*Salmonella* Typhimurium	1,4,[5],12:i:1,2	10	19
*Salmonella* Typhimurium monophasic	1,4,[5],12:i:-	5	34
*Salmonella* Hadar	6,8:z10:e,n,x	3	33
*Salmonella* Infantis	6,7,14:r:1,5	1	32

**Table 4:** Conjugative transfer of *tet(A)* and *tet(B)* genes from ambulatory *S. enterica* isolates.

Receptor	Donors	ARG	Conjugation Frequency	Transferred ARGs
*Escherichia coli* J53	S135 *S.* Hadar	*tet(A)*	3.5 × 10^−10^	-
	S136 *S.* Hadar	*tet(A)*	2.36 × 10^−10^	-
	S29 *S.* Hadar	*tet(A)*	3.3 × 10^−6^	-
	S96 *S.* 1,4,[5],12:i:-	*tet(B)*	4.66 × 10^−1^	-
	S107 *S.* 1,4,[5],12:i:-	*tet (B)*	3.33 × 10^−1^	-
	S149 *S.* 1,4,[5],12:i:-	*tet(B)*	3.5	-

PCR was performed to detect tetracycline resistance genes in transconjugants.

**Table 5:** Summary of antibiotics used for susceptibility testing of *Salmonella* spp. strains.

Class	Antibiotic(s) Tested
β-lactams inhibitors	Amoxicillin-clavulanate
	Ampicillin-sulbactam
	Piperacillin-tazobactam
Monobactams	Aztreonam
	Imipenem
Carbapenems	Ertapenem
	Meropenem
	Doripenem
Folate Pathway Antagonists	Trimethoprim-sulfamethoxazole
	Ampicillin
Penicillins	Piperacillin
Tetracyclines	Tetracycline
Quinolones and Fluoroquinolones	Ciprofloxacin Ofloxacin Levofloxacin Moxifloxacin
	Cefepime
Cephalosporins	Cefotaxime
	Ceftazidime
	Ceftriaxone
	Cefpodoxime
Other	Tigecycline
	Chloramphenicol
	Fosfomycin

## Data Availability

The *Salmonella* isolates, datasets containing experimental results, and prior characterisation data for AgNPsol are available upon request from the authors.

## Supplementary Materials

The following supporting information can be downloaded at https://www.mdpi.com/article/10.3390/antibiotics14010046/s1: Figures S1 and S2: Electrophoresis gel for *invA* gene detection in selected *Salmonella* spp. strains; Figure S3: Electrophoresis gel for *pldA* and *helD* genes detection in selected *Salmonella* spp. strains; Figure S4: Electrophoresis gel for *spvC* gene detection in selected *Salmonella* spp. strains; Figure S5: Electrophoresis gel for *sul1* and *sul2* genes detection in selected *Salmonella* spp. strains; Figure S6: Electrophoresis gel for *qnrA* and *qnrB* genes detection in selected *Salmonella* spp. strains; Figure S7: Electrophoresis gel for *bla*_TEM_ and *bla*_KPC_ genes detection in selected *Salmonella* spp. strains; Figure S8. Transconjugants colony-forming units aspect on MacConkey agar supplementyed wit sodium azide (150 mg/L) and tetracycline (8 mg/L); Figure S9: Disk diffusion screening assays of the antimicrobial activity of the Ag2NP and Ag3NPsol against *Salmonella enterica* strains; Table S1: Primer sequences for antibiotic resistance and virulence markers detection; Table S2: Antibiotic resistance profiles of *Salmonella* spp. strains; Table S3: Intermediate profiles to different antibiotics of *Salmonella* strains; Table S4: Susceptibility profiles to different antibiotics of *Salmonella* strains; Table S5: Relative propensity for biofilm formation in *Salmonella* spp. strains; Table S6: Potentially significant correlations found between ARGs and VMs present in the samples; Table S7: The diverse virulence strategies of *S. enterica* clones, particularly the widespread ST19 clone, in outpatient infections; Table S8: The diameter of the inhibition zone of AgNPsol against *Salmonella* spp. strains; Table S9: The MIC and the corresponding MIC/2 and MIC/4 values of AgNPsol for PICA determination in the selected *S. enterica* strains. Supplementary File S1.
