# Supplementary material for: Emerging Resistance and Virulence Patterns in Salmonella enterica: Insights into Silver Nanoparticles as an Antimicrobial Strategy
Source: Antibiotics (Basel). 2025 Jan 7;14(1):46. doi: 10.3390/antibiotics14010046 (PMC11762817; doi:10.3390/antibiotics14010046)
Supplement: Supplementary file 1 [file antibiotics-14-00046-s001.zip › Supplementary Materials_Salmonella paper-02.01.2025.pdf]

antibiotics

MDPI

Supplementary Material

Emerging Resistance and Virulence Patterns in *Salmonella enterica*: Insights into Silver Nanoparticles as an Antimicrobial Strategy

Irina Gheorghe-Barbu[1,2†], Ilda Czobor Barbu[1,2*], Rareș-Ionuț Dragomir[1,2†], Ioana Cristina Marinaș[2], Miruna Silvia Stan[2,3], Radu Pericleanu[1,2], Andreea Ștefania Dumbravă[1,2], Liviu-Iulian Rotaru[1,4], Simona Paraschiv[4,6], Leontina Mirela Bănică[4,6], Ionuț Pecete[5], Dan Oțelea[4], Violeta Corina Cristea[6], Mircea Ioan Popa[6,7], Marilena Monica Țânțu[8], Marius Surleac[2,4]

Supplementary Table S1. Primer sequences for antibiotic resistance and virulence markers detection.

A. Primers for antibiotic resistance markers and integrase gene detection.

ARGs	Primer sequences	Amplicon size (bp)	Amplification program (second amplification cycle)	References
**β-lactam antibiotic resistance genes**				
bla$_{\text{TEM}}$	TEM-F: ATA AAA TTC TTG AAG AC<br>TEM-R: TTA CCA ATG CTT AAT CA	1080	30x (95° – 30 sec, 52° – 40 sec, 72° – 70 sec)	[122]
bla$_{\text{SHV}}$	SHV-F: TGG TTA TGC GTT ATA TTC GCC<br>SHV-R: GGT TAG CGT TGC CAG TGC T	868	30x (95° – 30 sec, 56° – 40 sec, 72° – 60 sec)	
bla$_{\text{CTX-M}}$	CTX-M-F: TCG TCT CTT CCA GAA TAA GG<br>CTX-M-R: AAG GAG AAC CAG GAA CCA CG	754	30x (95° – 30 sec, 56° – 40 sec, 72° – 60 sec)	[123]
bla$_{\text{VIM}}$	VIM-F: GAT GGT GTT TGG TCG CAT A<br>VIM-R: CGA ATG CGC AGC ACC AG	389	30x (95° – 30 sec, 55° – 40 sec, 72° – 50 sec)	[124]
bla$_{\text{IMP}}$	IMP-F-GGAATAGAGTGGCTTAAYTCTC<br>IMP-R-GGTTTAAYAAAACAACCACC	232		

*bla*NDM	NDM-F: GCA GCT TGT CGG CCA TGC GG GC<br>NDM-R:GGT CGC GAA GCT GAG CAC CGC AT	621	30x (95° – 30 sec, 55° – 40 sec, 72° – 50 sec)	
*bla*OXA-48	OXA-48-F: GCGTGGTTAAGGATGAACAC<br>OXA-48-R: CATCAAGTTCAACCCAACCG	438	30x(95°C–30 sec, 52°C – 40 sec, 72-50 sec)	
**tetracycline resistance genes**				
tet(A)	tet(A)-F: GCGCGATCTGGTTCACTCG<br>tet(A)-R: AGTCGACAGYRGCGCCGGC	164	25x(94°C-5 sec, 61°C-30 sec, 61°C-30 sec)	[125]
tet(B)	tet(B)-F: TACGTGAATTTATTGCTTCGG<br>tet(B)-R: ATACAGCATCCAAAGCGCAC	206		
tet(C)	tet(C)-F: GCGGGATATCGTCCATTCCG<br>tet(C)-R: GCGTAGAGGATCCACAGGACG	207		
tet(D)	tet(D)-F: GGAATATCTCCCGGAAGCGG<br>tet(D)-R: CACATTGGACAGTGCCAGCAG	187		
**sulphonamides resistance genes**				
sul 1	sul1-F: CGGCGTGGGCTACCTGAACG<br>sul 1-R: GCCGATCGCGTGAAGTTCCG	432	30x(94°C-15 sec, 69°C-30 sec, 72°C-60 sec);	[126]
sul 2	sul 2-F: CGGCATCGTCAACATAACC<br>sul 2-R: GTGTGCGGATGAAGTCAG	293		
dfrA1-aadA1	dfrA1-aadA1-F: AGCATTACCCAACCGAAAGT<br>dfrA1-aadA1-R: TGTCAGCAAGATAGCCAGAT	60	30x(94°C-30 s, 60°C-40 s, 72°C-1 min)	
**quinolones resistance genes**				
qnrA	qnrA-F: AGAGGATTTCTCACGCCAGG<br>qnrA-R: TGCCAGGCACAGATCTTGAC	580	35x (95°C-1 min, 54°C-1 min, 72°C-1 min)	[127]
qnrB	qnrB-F: GGMATHGAAATTCGCCACTG<br>qnrB-R: TTTGCYGYYCGCCAGTCGAA	264		

B. Primers for virulence markers

Gene	Primer sequences	Amplicon	Amplification program	References

		size (bp)	(second amplification cycle)	
**virulence genes**				
spvC	spvC-F: ACTCCTTGCACAACCAAATGCGGA<br>spvC -R: TGTCTTCTGCATTTCGCCACCATCA	571	35x(94 °C-30 sec, 64°C-30 sec, 72°C 30 sec)	[128]
invA	invA -F: ACAGTGCTCGTTTACGACCTGAAT<br>invA -R: AGACGACTGGTACTGATCGATAAT	244		[129]
pldA	pldA -F: CAGGGCTGGTTGTTGCCGGT<br>pldA -R: ACGCCACAGCGGAAATG	284	30x(95°C-1 min, 60°C-40sec, 72°C 1 min)	[129]
helD	helD -F: GGTTGCTGGCGCGTGGTGAA<br>helD -R: GCGTGAGGCAAGACGACGCT	370		

Supplementary Table S2. Antibiotic resistance profiles of *Salmonella* spp. strains

Strain code	Amoxicillin-Clavulanate	Ampicillin /Sulbactam	Ampicillin	Aztreonam	Cefepime	Cefotaxime	Cefpodoxime	Ceftazidime	Ceftriaxone	Chloramphenicol	Ciprofloxacin	Ertapenem	Fosfomycin	Moxifloxacin	Ofloxacin	Piperacillin/Tazobactam	Piperacillin	Tetracycline	Trimethoprim/Sulfamethoxazole	Total general
2																		1		1
9		1	1	1													1	1		5
10		1	1														1	1		4
11																		1		1
12																		1		1
15		1	1														1			3
18		1	1							1							1			4
21		1	1												1		1		1	5
23			1	1	1	1	1	1	1								1			8
24				1																1
28																		1		1
29																		1		1
33															1			1	1	3
34		1	1														1			3
41		1	1	1													1	1		5
50							1								1			1	1	4
51										1										1
52																		1		1
58		1	1														1			3
61		1	1														1		1	4
63		1	1														1			3
67		1	1														1			3
69		1	1														1			3
70																		1	1	2
73	1	1	1							1							1	1		5
75				1			1	1	1									1		5
77															1			1	1	3
80		1	1							1							1	1	1	6
82		1	1							1							1		1	5
88		1	1														1	1		4
93		1								1									1	3
96		1	1														1	1		4

Strain code	Amoxicillin-Clavulanate	Ampicillin /Sulbactam	Ampicillin	Aztreonam	Cefepime	Cefotaxime	Cefpodoxime	Ceftazidime	Ceftriaxone	Chloramphenicol	Ciprofloxacin	Ertapenem	Fosfomycin	Moxifloxacin	Ofloxacin	Piperacillin/Tazobactam	Piperacillin	Tetracycline	Trimethoprim/Sulfamethoxazole	Total general
97		1	1	1													1	1		5
98														1				1		2
99							1											1	1	3
101		1	1														1	1		4
103		1	1							1							1	1		5
106																			1	1
107	1	1	1														1	1		5
109		1	1														1	1		4
110										1				1	1			1		4
111		1	1														1	1		4
112											1									1
115				1																1
118		1	1														1			3
121															1			1		2
123				1																1
124				1																1
125				1																1
126				1				1												2
129		1	1							1							1		1	5
132		1	1							1							1	1	1	6
133				1																1
135														1				1		2
136														1				1		2
137															1			1		2
139		1	1														1	1		4
144				1																1
146		1	1												1		1		1	5
147		1	1														1	1		4
148		1	1														1		1	4
149		1	1														1	1		4
150												1								1
152																			1	1
162		1	1							1							1	1	1	6
165		1	1														1	1		4
170		1	1							1							1		1	5
171														1	1			1	1	4
172		1	1														1	1		4
175		1	1							1							1		1	5
176		1	1														1	1		4
177	1	1	1													1	1	1		6
178	1	1	1													1	1	1		6
180		1	1														1	1	1	5
181																		1		1
182																		1		1
183				1														1		2
189				1																1
190							1								1			1	1	4
195	1			1						1			1							4

199			1														1	1		3
200															1			1	1	3
203																		1		1
207				1											1			1	1	4
209			1														1			2
214		1	1														1	1		4
217		1	1														1	1		4
219		1	1														1	1		4
222				1														1		2
227										1					1			1		3
228																		1		1
229			1														1	1		3
230																		1		1
232										1										1
234		1	1														1	1	1	5
235															1			1	1	3
236		1	1														1	1		4
238		1	1														1	1		4
240																		1		1
241	1	1	1														1	1		5
242														1	1					2
247	1	1	1			1	1		1	1										7
248		1	1														1	1		4
250		1	1														1			3
252		1	1											1	1		1	1		6
254															1			1		2
255				1																1
256		1	1							1					1		1	1	1	7
259		1	1														1			3
268		1	1														1	1		4
269		1	1														1	1		4
270																		1		1
271																		1		1
274														1				1		2
275		1	1														1	1		4
276				1																1
280		1	1														1			3
284		1	1	1			1	1				1					1			7
286	1																			1
287	1		1																	2
291		1	1														1			3
293		1	1	1	1	1	1	1	1								1			9
294		1	1														1	1		4
295		1	1														1			3
296		1	1							1				1	1		1	1	1	8
297																			1	1
298				1	1							1						1		4
302														1	1			1		3
304		1	1														1			3
305			1	1	1	1	1	1	1								1	1		9
307		1	1														1	1		4
309		1	1														1			3
Total general	9	67	72	24	4	4	9	6	5	19	1	3	1	10	20	2	70	81	29	

Legend: Value 1 (red colour) was used for resistant strains to the following antibiotics: Amoxicillin-Clavulanate (MIC > 8/4 µg/mL); Ampicillin /Sulbactam (MIC >8/4 µg/mL); Ampicillin (MIC >8 µg/mL); Aztreonam (MIC =16 µg/mL); Cefepime (MIC = >8 µg/mL); Cefotaxime (MIC =16 µg/mL); Cefpodoxime (MIC >1 µg/mL); Ceftazidime (MIC > 16 µg/mL); Ceftriaxone (MIC =2 µg/mL); Chloramphenicol (MIC >8 µg/mL); Ciprofloxacin (MIC > 2 µg/mL); Ertapenem (MIC >1 µg/mL); Fosfomycin (MIC > 256 µg/mL); Moxifloxacin (MIC >1 µg/mL); Ofloxacin (MIC >1 µg/mL); Piperacillin/Tazobactam (MIC > 128/4 µg/mL); Piperacillin (MIC >16 µg/mL); Tetracycline (MIC >8 µg/mL); Trimethoprim/Sulfamethoxazole (MIC > 4/76 µg/mL).

Supplementary Table S3. Intermediate profiles to different antibiotics of *Salmonella* strains.

Strain code	Cefepime	Cefotaxime	Ceftazidime	Ceftriaxone	Ciprofloxacin	Doripenem	Ertapenem	Levofloxacin	Moxifloxacin	Ofloxacin	Tetracycline	Tigecycline	Total general
2	1												1
21									1				1
23										1			1
29									1	1			2
50		1							1			1	3
53				1									1
57										1			1
59										1			1
61									1	1			2
75	1	1											2
76										1			1
77									1			1	2
90										1			1
98										1			1
99					1				1	1		1	4
113										1			1
120										1			1
121					1				1			1	3
124				1									1
125				1									1
126				1									1
133									1	1			2
135										1			1
136										1			1
137					1				1				2
139							1						1
144		1											1
146									1				1
148									1	1			2
164				1									1
171					1			1					2
182	1												1
183												1	1
190									1				1
195						1							1
196										1			1
200					1				1			1	3
207		1	1						1			1	4
222												1	1
241										1			1
254					1				1			1	3
256									1				1
262										1			1
270									1				1
274										1			1
286										1			1
291											1		1
294							1						1
296					1								1
Total	3	4	1	5	7	1	2	1	17	20	1	9	

Supplementary Table S4. Susceptibility profiles to different antibiotics of *Salmonella* strains.

Legend: Value 1 (yellow colour) was used for intermediate profile to the following antibiotics: Cefepime (MIC = >8 μg/mL); Cefotaxime (MIC =2 μg/mL); Ceftazidime (MIC =2 μg/mL); Ceftriaxone (MIC =2 μg/mL); Ciprofloxacin (MIC =1 μg/mL); Ertapenem (MIC =1 μg/mL); Levofloxacin (MIC =2 μg/mL); Moxifloxacin (MIC =1 μg/mL); Ofloxacin (MIC =1 μg/mL); Tetracycline (MIC = 8 μg/mL);Tigecycline (MIC =2 μg/mL).

Legend: Value 1 (green colour) was used for susceptible strains to the following antibiotics: Amoxicillin-Clavulanate (MIC <=2/1 µg/mL); Ampicillin /Sulbactam (MIC <=1/.5 µg/mL); Ampicillin (MIC =1 µg/mL); Aztreonam (MIC <=1 µg/mL); Cefepime (MIC <=0.5 µg/mL); Cefotaxime (MIC <=0.5 µg/mL); Cefpodoxime (MIC <=0.5 µg/mL); Ceftazidime (MIC <=0.5 µg/mL); Ceftriaxone (MIC <=0.5 µg/mL); Chloramphenicol (MIC <=8 µg/mL); Ciprofloxacin (MIC <=0.5 µg/mL); Doripenem (MIC <=1 µg/mL); Ertapenem (MIC <=0.5 µg/mL); Imipenem (MIC <=1 µg/mL); Meropenem (MIC <=0.5 µg/mL); Levofloxacin (MIC <=1 µg/mL); Fosfomycin (MIC <=32 µg/mL); Moxifloxacin (MIC <=0.5 µg/mL); Ofloxacin (MIC <=0.5 µg/mL); Piperacillin/Tazobactam (MIC <=4 µg/mL); Piperacillin (MIC <=4 µg/mL); Tetracycline (MIC <=4 µg/mL); Tigecycline (MIC= 0.5 µg/mL); Trimethoprim/Sulfamethoxazole (MIC <=2/38 µg/mL).

Strain code	Amoxicillin-Clavulanate	Ampicillin/Sulbactam	Ampicillin	Aztreonam	Cefepime	Cefotaxime	Cefpodoxime	Ceftazidime	Ceftriaxone	Chloramphenicol	Ciprofloxacin	Doripenem	Ertapenem	Fosfomycin	Imipenem	Levofloxacin	Meropenem	Moxifloxacin	Ofloxacin	Piperacillin/Tazobactam	Piperacillin	Tetracycline	Tigecycline	Trimethoprim/Sulfamethoxazole	Total general
1	1	1	1	1	1	1	1	1	1	1	1	1	1	1	1	1	1	1	1	1	1	1	1	1	24
2	1	1	1	1		1	1	1	1	1	1	1	1	1	1	1	1	1	1	1	1		1	1	22
3	1	1	1	1	1	1	1	1	1	1	1	1	1	1	1	1	1	1	1	1	1	1	1	1	24
4	1	1	1	1	1	1	1	1	1	1	1	1	1	1	1	1	1	1	1	1	1	1	1	1	23
5	1	1	1	1	1	1	1	1	1	1	1	1	1	1	1	1	1	1	1	1	1	1	1	1	23
6	1	1	1	1	1	1	1	1	1	1	1	1	1	1	1	1	1	1	1	1	1	1	1	1	23
7	1	1	1	1	1	1	1	1	1	1	1	1	1	1	1	1	1	1	1	1	1	1	1	1	23
8	1	1	1	1	1	1	1	1	1	1	1	1	1	1	1	1	1	1	1	1	1	1	1	1	23
9	1				1	1	1	1	1	1	1	1	1	1	1	1	1	1	1	1			1	1	18
10	1			1	1	1	1	1	1	1	1	1	1	1	1	1	1	1	1	1			1	1	20
11	1	1	1	1	1	1	1	1	1	1	1	1	1	1	1	1	1	1	1	1	1		1	1	23
12	1	1	1	1	1	1	1	1	1	1	1	1	1	1	1	1	1	1	1	1	1		1	1	23
13	1	1	1	1	1	1	1	1	1	1	1	1	1	1	1	1	1	1	1	1	1	1	1	1	24
14	1	1	1	1	1	1	1	1	1	1	1	1	1	1	1	1	1	1	1	1	1	1	1	1	24
15	1			1	1	1	1	1	1	1	1	1	1	1	1	1	1	1	1	1		1	1	1	21
16	1	1	1	1	1	1	1	1	1	1	1	1	1	1	1	1	1	1	1	1	1	1	1	1	24
17	1	1	1	1	1	1	1	1	1	1	1	1	1	1	1	1	1	1	1	1	1	1	1	1	24
18	1			1	1	1	1	1	1		1	1	1	1	1	1	1	1	1	1		1	1	1	20
19	1	1	1	1	1	1	1	1	1	1	1	1	1	1	1	1	1	1	1	1	1	1	1	1	24
20	1	1	1	1	1	1	1	1	1	1	1	1	1	1	1	1	1	1	1	1	1	1	1	1	24
21	1			1	1	1	1	1	1	1	1	1	1	1	1	1	1			1		1	1		18
22	1	1	1	1	1	1	1	1	1	1	1	1	1	1	1	1	1	1	1	1	1	1	1	1	24
23	2	2	1	1	1	1	1	1	1	2	2	2	2	2	2	2	2	2	1	2	1	2	2	2	39
24	1	1	1		1	1	1	1	1	1	1	1	1	1	1	1	1	1	1	1	1	1	1	1	23
25	1	1	1	1	1	1	1	1	1	1	1	1	1	1	1	1	1	1	1	1	1	1	1	1	24
26	1	1	1	1	1	1	1	1	1	1	1	1	1	1	1	1	1	1	1	1	1	1	1	1	24
27	1	1	1	1	1	1	1	1	1	1	1	1	1	1	1	1	1	1	1	1	1	1	1	1	24
28	1	1	1	1	1	1	1	1	1	1	1	1	1	1	1	1	1	1	1	1	1		1	1	23
29	1	1	1	1	1	1	1	1	1	1	1	1	1	1	1	1	1			1	1		1	1	21
30	1	1	1	1	1	1	1	1	1	1	1	1	1	1	1	1	1	1	1	1	1	1	1	1	24
31	1	1	1	1	1	1	1	1	1	1	1	1	1	1	1	1	1	1	1	1	1	1	1	1	24
32	1	1	1	1	1	1	1	1	1	1	1	1	1	1	1	1	1	1	1	1	1	1	1	1	24
33	1	1	1	1	1	1	1	1	1	1	1	1	1	1	1	1	1	1		1	1		1		21
34	1			1	1	1	1	1	1	1	1	1	1	1	1	1	1	1	1	1		1	1	1	20
35	1	1	1	1	1	1	1	1	1	1	1	1	1	1	1	1	1	1	1	1	1	1	1	1	23
36	1	1	1	1	1	1	1	1	1	1	1	1	1	1	1	1	1	1	1	1	1	1	1	1	23
38	1	1	1	1	1	1	1	1	1	1	1	1	1	1	1	1	1	1	1	1	1	1	1	1	23
39	1	1	1	1	1	1	1	1	1	1	1	1	1	1	1	1	1	1	1	1	1	1	1	1	23
40	1	1	1	1	1	1	1	1	1	1	1	1	1	1	1	1	1	1	1	1	1	1	1	1	23
41	1				1	1	1	1	1	1	1	1	1	1	1	1	1	1	1	1			1	1	18
42	1	1	1	1	1	1	1	1	1	1	1	1	1	1	1	1	1	1	1	1	1	1	1	1	23
43	1	1	1	1	1	1	1	1	1	1	1	1	1	1	1	1	1	1	1	1	1	1	1	1	23
44	1	1	1	1	1	1	1	1	1	1	1	1	1	1	1	1	1	1	1	1	1	1	1	1	23
45	1	1	1	1	1	1	1	1	1	1	1	1	1	1	1	1	1	1	1	1	1	1	1	1	23
46	1	1	1	1	1	1	1	1	1	1	1	1	1	1	1	1	1	1	1	1	1	1	1	1	23
47	1	1	1	1	1	1	1	1	1	1	1	1	1	1	1	1	1	1	1	1	1	1	1	1	23
48	1	1	1	1	1	1	1	1	1	1	1	1	1	1	1	1	1	1	1	1	1	1	1	1	23
49	1	1	1	1	1	1	1	1	1	1	1	1	1	1	1	1	1	1	1	1	1	1	1	1	23
50	1	1	1	1	1			1	1	1	1	1	1	1	1	1	1			1	1				16
51	1	1	1	1	1	1	1	1	1		1	1	1	1	1	1	1	1	1	1	1	1	1	1	22
52	1	1	1	1	1	1	1	1	1	1	1	1	1	1	1	1	1	1	1	1	1		1	1	22
53	1	1	1	1	1	1	1	1		1	1	1	1	1	1	1	1	1	1	1	1	1	1	1	22
54	1	1	1	1	1	1	1	1	1	1	1	1	1	1	1	1	1	1	1	1	1	1	1	1	23
55	1	1	1	1	1	1	1	1	1	1	1	1	1	1	1	1	1	1	1	1	1	1	1	1	23
56	1	1	1	1	1	1	1	1	1	1	1	1	1	1	1	1	1	1	1	1	1	1	1	1	23
57	1	1	1	1	1	1	1	1	1	1	1	1	1	1	1	1	1	1		1	1	1	1	1	22
58	1			1	1	1	1	1	1	1	1	1	1	1	1	1	1	1	1	1		1	1	1	20
59	1	1	1	1	1	1	1	1	1	1	1	1	1	1	1	1	1	1		1	1	1	1	1	22
60	1	1	1	1	1	1	1	1	1	1	1	1	1	1	1	1	1	1	1	1	1	1	1	1	23
61	1			1	1	1	1	1	1	1	1	1	1	1	1	1	1			1		1	1		17
62	1	1	1	1	1	1	1	1	1	1	1	1	1	1	1	1	1	1	1	1	1	1	1	1	23
63	1			1	1	1	1	1	1	1	1	1	1	1	1	1	1	1	1	1		1	1	1	20
64	1	1	1	1	1	1	1	1	1	1	1	1	1	1	1	1	1	1	1	1	1	1	1	1	23
65	1	1	1	1	1	1	1	1	1	1	1	1	1	1	1	1	1	1	1	1	1	1	1	1	23
66	1	1	1	1	1	1	1	1	1	1	1	1	1	1	1	1	1	1	1	1	1	1	1	1	23
67	1			1	1	1	1	1	1	1	1	1	1	1	1	1	1	1	1	1		1	1	1	20
68	1	1	1	1	1	1	1	1	1	1	1	1	1	1	1	1	1	1	1	1	1	1	1	1	23
69	1			1	1	1	1	1	1	1	1	1	1	1	1	1	1	1	1	1		1	1	1	20
70	1	1	1	1	1	1	1	1	1	1	1	1	1	1	1	1	1	1	1	1	1		1		21
71	1	1	1	1	1	1	1	1	1	1	1	1	1	1	1	1	1	1	1	1	1	1	1	1	23
72	1	1	1	1	1	1	1	1	1	1	1	1	1	1	1	1	1	1	1	1	1	1	1	1	23
73				1	1	1	1	1	1		1	1	1	1	1	1	1	1	1	1			1	1	18
74	1	1	1	1	1	1	1	1	1	1	1	1	1	1	1	1	1	1	1	1	1	1	1	1	23
75	1	1	1							1	1	1	1	1	1	1	1	1	1	1	1		1	1	16
76	1	1	1	1	1	1	1	1	1	1	1	1	1	1	1	1	1	1		1	1	1	1	1	22
77	1	1	1	1	1	1	1	1	1	1	1	1	1	1	1	1	1			1	1				18
78	1	1	1	1	1	1	1	1	1	1	1	1	1	1	1	1	1	1	1	1	1	1	1	1	23
79	1	1	1	1	1	1	1	1	1	1	1	1	1	1	1	1	1	1	1	1	1	1	1	1	23
80	1			1	1	1	1	1	1		1	1	1	1	1	1	1	1	1	1			1		17
81	1	1	1	1	1	1	1	1	1	1	1	1	1	1	1	1	1	1	1	1	1	1	1	1	23
82	1			1	1	1	1	1	1		1	1	1	1	1	1	1	1	1	1		1	1		18
83	1	1	1	1	1	1	1	1	1	1	1	1	1	1	1	1	1	1	1	1	1	1	1	1	23
84	1	1	1	1	1	1	1	1	1	1	1	1	1	1	1	1	1	1	1	1	1	1	1	1	23
85	1	1	1	1	1	1	1	1	1	1	1	1	1	1	1	1	1	1	1	1	1	1	1	1	23
86	1	1	1	1	1	1	1	1	1	1	1	1	1	1	1	1	1	1	1	1	1	1	1	1	23
87	1	1	1	1	1	1	1	1	1	1	1	1	1	1	1	1	1	1	1	1	1	1	1	1	23
88	1			1	1	1	1	1	1	1	1	1	1	1	1	1	1	1	1	1			1	1	19
89	1	1	1	1	1	1	1	1	1	1	1	1	1	1	1	1	1	1	1	1	1	1	1	1	23
90	1	1	1	1	1	1	1	1	1	1	1	1	1	1	1	1	1	1		1	1	1	1	1	22
91	1	1	1	1	1	1	1	1	1	1	1	1	1	1	1	1	1	1	1	1	1	1	1	1	23
92	1	1	1	1	1	1	1	1	1	1	1	1	1	1	1	1	1	1	1	1	1	1	1	1	23
93	1										1	1	1	1	1	1	1	1	1	1		1	1		12
94	1	1	1	1	1	1	1	1	1	1	1	1	1	1	1	1	1	1	1	1	1	1	1	1	23
95	1	1	1	1	1	1	1	1	1	1	1	1	1	1	1	1	1	1	1	1	1	1	1	1	23
96	1			1	1	1	1	1	1	1	1	1	1	1	1	1	1	1	1	1			1	1	19
97	1				1	1	1	1	1	1	1	1	1	1	1	1	1	1	1	1			1	1	18
98	1	1	1	1	1	1	1	1	1	1	1	1	1	1	1	1	1			1	1		1	1	20
99	1	1	1	1	1	1		1	1	1		1	1	1	1	1	1			1	1				16
100	1	1	1	1	1	1	1	1	1	1	1	1	1	1	1	1	1	1	1	1	1	1	1	1	24
101	1			1	1	1	1	1	1	1	1	1	1	1	1	1	1	1	1	1			1	1	20
102	1	1	1	1	1	1	1	1	1	1	1	1	1	1	1	1	1	1	1	1	1	1	1	1	24
103	1			1	1	1	1	1	1		1	1	1	1	1	1	1	1	1	1			1	1	19
104	1	1	1	1	1	1	1	1	1	1	1	1	1	1	1	1	1	1	1	1	1	1	1	1	24
105	1	1	1	1	1	1	1	1	1	1	1	1	1	1	1	1	1	1	1	1	1	1	1	1	24
106	1	1	1	1	1	1	1	1	1	1	1	1	1	1	1	1	1	1	1	1	1	1	1		23
107				1	1	1	1	1	1	1	1	1	1	1	1	1	1	1	1	1			1	1	19
108	1	1	1	1	1	1	1	1	1	1	1	1	1	1	1	1	1	1	1	1	1	1	1	1	24
109	1			1	1	1	1	1	1	1	1	1	1	1	1	1	1	1	1	1			1	1	20
110	1	1	1	1	1	1	1	1	1		1	1	1	1	1	1	1			1	1		1	1	20
111	1			1	1	1	1	1	1	1	1	1	1	1	1	1	1	1	1	1			1	1	20
112	1	1	1	1	1	1	1	1	1	1		1	1	1	1	1	1	1	1	1	1	1	1	1	23
113	1	1	1	1	1	1	1	1	1	1	1	1	1	1	1	1	1	1		1	1	1	1	1	23
114	1	1	1	1	1	1	1	1	1	1	1	1	1	1	1	1	1	1	1	1	1	1	1	1	24
115	1	1	1		1	1	1	1	1	1	1	1	1	1	1	1	1	1	1	1	1	1	1	1	23
116	1	1	1	1	1	1	1	1	1	1	1	1	1	1	1	1	1	1	1	1	1	1	1	1	24
117	1	1	1	1	1	1	1	1	1	1	1	1	1	1	1	1	1	1	1	1	1	1	1	1	24
118	1			1	1	1	1	1	1	1	1	1	1	1	1	1	1	1	1	1		1	1	1	21
119	1	1	1	1	1	1	1	1	1	1	1	1	1	1	1	1	1	1	1	1	1	1	1	1	24
120	1	1	1	1	1	1	1	1	1	1	1	1	1	1	1	1	1	1		1	1	1	1	1	23
121	1	1	1	1	1	1	1	1	1	1		1	1	1	1	1	1			1	1			1	19
122	1	1	1	1	1	1	1	1	1	1	1	1	1	1	1	1	1	1	1	1	1	1	1	1	24
123	1	1	1		1	1	1	1	1	1	1	1	1	1	1	1	1	1	1	1	1	1	1	1	23
124	1	1	1		1	1	1	1		1	1	1	1	1	1	1	1	1	1	1	1	1	1	1	22
125	1	1	1		1	1	1	1		1	1	1	1	1	1	1	1	1	1	1	1	1	1	1	22
126	1	1	1		1	1	1			1	1	1	1	1	1	1	1	1	1	1	1	1	1	1	21
127	1	1	1	1	1	1	1	1	1	1	1	1	1	1	1	1	1	1	1	1	1	1	1	1	24
128	1	1	1	1	1	1	1	1	1	1	1	1	1	1	1	1	1	1	1	1	1	1	1	1	24
129	1			1	1	1	1	1	1		1	1	1	1	1	1	1	1	1	1		1	1		19
130	1	1	1	1	1	1	1	1	1	1	1	1	1	1	1	1	1	1	1	1	1	1	1	1	24
131	1	1	1	1	1	1	1	1	1	1	1	1	1	1	1	1	1	1	1	1	1	1	1	1	24
132	1			1	1	1	1	1	1		1	1	1	1	1	1	1	1	1	1			1		18
133	1	1	1		1	1	1	1	1	1	1	1	1	1	1	1	1			1	1	1	1	1	21
134	1	1	1	1	1	1	1	1	1	1	1	1	1	1	1	1	1	1	1	1	1	1	1	1	24
135	1	1	1	1	1	1	1	1	1	1	1	1	1	1	1	1	1			1	1		1	1	21
136	1	1	1	1	1	1	1	1	1	1	1	1	1	1	1	1	1			1	1		1	1	21
137	1	1	1	1	1	1	1	1	1	1		1	1	1	1	1	1			1	1		1	1	20
138	1	1	1	1	1	1	1	1	1	1	1	1	1	1	1	1	1	1	1	1	1	1	1	1	24
139	1			1	1	1	1	1	1	1	1	1		1	1	1	1	1	1	1			1	1	19
140	1	1	1	1	1	1	1	1	1	1	1	1	1	1	1	1	1	1	1	1	1	1	1	1	24
141	1	1	1	1	1	1	1	1	1	1	1	1	1	1	1	1	1	1	1	1	1	1	1	1	24
142	1	1	1	1	1	1	1	1	1	1	1	1	1	1	1	1	1	1	1	1	1	1	1	1	24
143	1	1	1	1	1	1	1	1	1	1	1	1	1	1	1	1	1	1	1	1	1	1	1	1	24
144	1	1	1		1		1	1	1	1	1	1	1	1	1	1	1	1	1	1	1	1	1	1	22
145	1	1	1	1	1	1	1	1	1	1	1	1	1	1	1	1	1	1	1	1	1	1	1	1	24
146	1			1	1	1	1	1	1	1	1	1	1	1	1	1	1			1		1	1		18
147	1			1	1	1	1	1	1	1	1	1	1	1	1	1	1	1	1	1			1	1	20
148	1			1	1	1	1	1	1	1	1	1	1	1	1	1	1			1		1	1		18
149	1			1	1	1	1	1	1	1	1	1	1	1	1	1	1	1	1	1			1	1	20
150	1	1	1	1	1	1	1	1	1	1	1	1		1	1	1	1	1	1	1	1	1	1	1	23
151	1	1	1	1	1	1	1	1	1	1	1	1	1	1	1	1	1	1	1	1	1	1	1	1	24
152	1	1	1	1	1	1	1	1	1	1	1	1	1	1	1	1	1	1	1	1	1	1	1		23
153	1	1	1	1	1	1	1	1	1	1	1	1	1	1	1	1	1	1	1	1	1	1	1	1	24
154	1	1	1	1	1	1	1	1	1	1	1	1	1	1	1	1	1	1	1	1	1	1	1	1	24
155	1	1	1	1	1	1	1	1	1	1	1	1	1	1	1	1	1	1	1	1	1	1	1	1	24
156	1	1	1	1	1	1	1	1	1	1	1	1	1	1	1	1	1	1	1	1	1	1	1	1	24
157	1	1	1	1	1	1	1	1	1	1	1	1	1	1	1	1	1	1	1	1	1	1	1	1	24
158	1	1	1	1	1	1	1	1	1	1	1	1	1	1	1	1	1	1	1	1	1	1	1	1	24
159	1	1	1	1	1	1	1	1	1	1	1	1	1	1	1	1	1	1	1	1	1	1	1	1	24
160	1	1	1	1	1	1	1	1	1	1	1	1	1	1	1	1	1	1	1	1	1	1	1	1	24
162	1			1	1	1	1	1	1		1	1	1	1	1	1	1	1	1	1			1		18
163	1	1	1	1	1	1	1	1	1	1	1	1	1	1	1	1	1	1	1	1	1	1	1	1	24
164	1	1	1	1	1	1	1	1		1	1	1	1	1	1	1	1	1	1	1	1	1	1	1	23
165	1			1	1	1	1	1	1	1	1	1	1	1	1	1	1	1	1	1			1	1	20
167	1	1	1	1	1	1	1	1	1	1	1	1	1	1	1	1	1	1	1	1	1	1	1	1	24
168	1	1	1	1	1	1	1	1	1	1	1	1	1	1	1	1	1	1	1	1	1	1	1	1	24
169	1	1	1	1	1	1	1	1	1	1	1	1	1	1	1	1	1	1	1	1	1	1	1	1	24
170	1			1	1	1	1	1	1		1	1	1	1	1	1	1	1	1	1		1	1		19
171	1	1	1	1	1	1	1	1	1	1		1	1	1	1		1			1	1		1		18
172	1			1	1	1	1	1	1	1	1	1	1	1	1	1	1	1	1	1			1	1	20
173	1	1	1	1	1	1	1	1	1	1	1	1	1	1	1	1	1	1	1	1	1	1	1	1	24
174	1	1	1	1	1	1	1	1	1	1	1	1	1	1	1	1	1	1	1	1	1	1	1	1	24
175	1			1	1	1	1	1	1		1	1	1	1	1	1	1	1	1	1		1	1		19
176	1			1	1	1	1	1	1	1	1	1	1	1	1	1	1	1	1	1			1	1	20
177				1	1	1	1	1	1	1	1	1	1	1	1	1	1	1	1				1	1	18
178				1	1	1	1	1	1	1	1	1	1	1	1	1	1	1	1				1	1	18
179	1	1	1	1	1		1	1		1	1	1	1	1	1	1	1	1	1	1	1	1	1	1	22
180	1			1	1	1	1	1	1	1	1	1	1	1	1	1	1	1	1	1			1		19
181	1	1	1	1	1	1	1	1	1	1	1	1	1	1	1	1	1	1	1	1	1		1	1	23
182	1	1	1	1		1	1	1	1	1	1	1	1	1	1	1	1	1	1	1	1		1	1	22
183	1	1	1		1	1	1	1	1	1	1	1	1	1	1	1	1	1	1	1	1			1	21
184	1	1	1	1	1	1	1	1	1	1	1	1	1	1	1	1	1	1	1	1	1	1	1	1	24
185	1	1	1	1	1	1	1	1	1	1	1	1	1	1	1	1	1	1	1	1	1	1	1	1	24
186	1	1	1	1	1	1	1	1	1	1	1	1	1	1	1	1	1	1	1	1	1	1	1		23
187	1	1	1	1	1	1	1	1	1	1	1	1	1	1	1	1	1	1	1	1	1	1	1	1	24

188	1	1	1	1	1	1	1	1	1	1	1	1	1	1	1	1	1	1	1	1	1	1	1	1	24
189	1	1	1		1	1	1	1	1	1	1	1	1	1	1	1	1	1	1	1	1	1	1	1	23
190	1	1	1	1	1	1		1	1	1	1	1	1	1	1	1	1			1	1		1		19
191	1	1	1	1	1	1	1	1	1	1	1	1	1	1	1	1	1	1	1	1	1	1	1	1	24
192	1	1	1	1	1	1	1	1	1	1	1	1	1	1	1	1	1	1	1	1	1	1	1	1	24
193	1	1	1	1	1	1	1	1	1	1	1	1	1	1	1	1	1	1	1	1	1	1	1	1	24
194	1	1	1	1	1	1	1	1	1	1	1	1	1	1	1	1	1	1	1	1	1	1	1	1	24
195		1	1		1	1	1	1	1		1		1		1	1	1	1	1	1	1	1	1	1	19
196	1	1	1	1	1	1	1	1	1	1	1	1	1	1	1	1	1	1		1	1	1	1	1	23
197	1	1	1	1	1	1	1	1	1	1	1	1	1	1	1	1	1	1	1	1	1	1	1	1	24
198	1	1	1	1	1	1	1	1	1	1	1	1	1	1	1	1	1	1	1	1	1	1	1	1	24
199	1	1		1	1	1	1	1	1	1	1	1	1	1	1	1	1	1	1	1			1	1	21
200	1	1	1	1	1	1	1	1	1	1		1	1	1	1	1	1			1	1				18
201	1	1	1	1	1	1	1	1	1	1	1	1	1	1	1	1	1	1	1	1	1	1	1	1	24
202	1	1	1	1	1	1	1	1	1	1	1	1	1	1	1	1	1	1	1	1	1	1	1	1	24
203	1	1	1	1	1	1	1	1	1	1	1	1	1	1	1	1	1	1	1	1	1		1	1	23
204	1	1	1	1	1	1	1	1	1	1	1	1	1	1	1	1	1	1	1	1	1	1	1	1	24
205	1	1	1	1	1	1	1	1	1	1	1	1	1	1	1	1	1	1	1	1	1	1	1	1	24
206	1	1	1	1	1	1	1	1	1	1	1	1	1	1	1	1	1	1	1	1	1	1	1	1	24
207	1	1	1		1		1		1	1	1	1	1	1	1	1	1			1	1				16
208	1	1	1	1	1	1	1	1	1	1	1	1	1	1	1	1	1	1	1	1	1	1	1	1	24
209	1	1		1	1	1	1	1	1	1	1	1	1	1	1	1	1	1	1	1		1	1	1	22
210	1	1	1	1	1	1	1	1	1	1	1	1	1	1	1	1	1	1	1	1	1	1	1	1	24
211	1	1	1	1	1	1	1	1	1	1	1	1	1	1	1	1	1	1	1	1	1	1	1	1	24
212	1	1	1	1	1	1	1	1	1	1	1	1	1	1	1	1	1	1	1	1	1	1	1	1	24
213	1	1	1	1	1	1	1	1	1	1	1	1	1	1	1	1	1	1	1	1	1	1	1	1	24
214	1			1	1	1	1	1	1	1	1	1	1	1	1	1	1	1	1	1			1	1	20
215	1	1	1	1	1	1	1	1	1	1	1	1	1	1	1	1	1	1	1	1	1	1	1	1	24
216	1	1	1	1	1	1	1	1	1	1	1	1	1	1	1	1	1	1	1	1	1	1	1	1	24
217	1			1	1	1	1	1	1	1	1	1	1	1	1	1	1	1	1	1			1	1	20
218	1	1	1	1	1	1	1	1	1	1	1	1	1	1	1	1	1	1	1	1	1	1	1	1	24
219	1			1	1	1	1	1	1	1	1	1	1	1	1	1	1	1	1	1			1	1	20
220	1	1	1	1	1	1	1	1	1	1	1	1	1	1	1	1	1	1	1	1	1	1	1	1	24
221	1	1	1	1	1	1	1	1	1	1	1	1	1	1	1	1	1	1	1	1	1	1	1	1	24
222	1	1	1		1	1	1	1	1	1	1	1	1	1	1	1	1	1	1	1	1			1	21
223	1	1	1	1	1	1	1	1	1	1	1	1	1	1	1	1	1	1	1	1	1	1	1	1	24
224	1	1	1	1	1	1	1	1	1	1	1	1	1	1	1	1	1	1	1	1	1	1	1	1	24
225	1	1	1	1	1	1	1	1	1	1	1	1	1	1	1	1	1	1	1	1	1	1	1	1	24
226	1	1	1	1	1	1	1	1	1	1	1	1	1	1	1	1	1	1	1	1	1	1	1	1	24
227	1	1	1	1	1	1	1	1	1		1	1	1	1	1	1	1	1		1	1		1	1	21
228	1	1	1	1	1	1	1	1	1	1	1	1	1	1	1	1	1	1	1	1	1		1	1	23
229	1	1		1	1	1	1	1	1	1	1	1	1	1	1	1	1	1	1	1			1	1	21
230	1	1	1	1	1	1	1	1	1	1	1	1	1	1	1	1	1	1	1	1	1		1	1	23
231	1	1	1	1	1	1	1	1	1	1	1	1	1	1	1	1	1	1	1	1	1	1	1	1	24
232	1	1	1	1	1	1	1	1	1		1	1	1	1	1	1	1	1	1	1	1	1	1	1	23
233	1	1	1	1	1	1	1	1	1	1	1	1	1	1	1	1	1	1	1	1	1	1	1	1	24
234	1			1	1	1	1	1	1	1	1	1	1	1	1	1	1	1	1	1			1		19
235	1	1	1	1	1	1	1	1	1	1	1	1	1	1	1	1	1	1		1	1		1		21
236	1			1	1	1	1	1	1	1	1	1	1	1	1	1	1	1	1	1			1	1	20
237	1	1	1	1	1	1	1	1	1	1	1	1	1	1	1	1	1	1	1	1	1	1	1	1	24
238	1			1	1	1	1	1	1	1	1	1	1	1	1	1	1	1	1	1			1	1	20
239	1	1	1	1	1	1	1	1	1	1	1	1	1	1	1	1	1	1	1	1	1	1	1	1	24
240	1	1	1	1	1	1	1	1	1	1	1	1	1	1	1	1	1	1	1	1	1		1	1	23
241				1	1	1	1	1	1	1	1	1	1	1	1	1	1	1		1			1	1	18
242	1	1	1	1	1	1	1	1	1	1	1	1	1	1	1	1	1			1	1	1	1	1	22
243	1	1	1	1	1	1	1	1	1	1	1	1	1	1	1	1	1	1	1	1	1	1	1	1	24
244	1	1	1	1	1	1	1	1	1	1	1	1	1	1	1	1	1	1	1	1	1	1	1	1	24
245	1	1	1	1	1	1	1	1	1	1	1	1	1	1	1	1	1	1	1	1	1	1	1	1	24
246	1	1	1	1	1	1	1	1	1	1	1	1	1	1	1	1	1	1	1	1	1	1	1	1	24
247				1	1			1			1	1	1	1	1	1	1	1	1	1	1	1	1	1	17
248	1			1	1	1	1	1	1	1	1	1	1	1	1	1	1	1	1	1			1	1	20
249	1	1	1	1	1	1	1	1	1	1	1	1	1	1	1	1	1	1	1	1	1	1	1	1	24
250	1			1	1	1	1	1	1	1	1	1	1	1	1	1	1	1	1	1		1	1	1	21
251	1	1	1	1	1	1	1	1	1	1	1	1	1	1	1	1	1	1	1	1	1	1	1	1	24
252	1			1	1	1	1	1	1	1	1	1	1	1	1	1	1			1			1	1	18
253	1	1	1	1	1	1	1	1	1	1	1	1	1	1	1	1	1	1	1	1	1	1	1	1	24
254	1	1	1	1	1	1	1	1	1	1		1	1	1	1	1	1			1	1			1	19
255	1	1	1		1	1	1	1	1	1	1	1	1	1	1	1	1	1	1	1	1	1	1	1	23
256	1			1	1	1	1	1	1		1	1	1	1	1	1	1			1			1		16
257	1	1	1	1	1	1	1	1	1	1	1	1	1	1	1	1	1	1	1	1	1	1	1	1	24
258	1	1	1	1	1	1	1	1	1	1	1	1	1	1	1	1	1	1	1	1	1	1	1	1	24
259	1			1	1	1	1	1	1	1	1	1	1	1	1	1	1	1	1	1		1	1	1	21
260	1	1	1	1	1	1	1	1	1	1	1	1	1	1	1	1	1	1	1	1	1	1	1	1	24
261	1	1	1	1	1	1	1	1	1	1	1	1	1	1	1	1	1	1	1	1	1	1	1	1	24
262	1	1	1	1	1	1	1	1	1	1	1	1	1	1	1	1	1	1		1	1	1	1	1	23
263	1	1	1	1	1	1	1	1	1	1	1	1	1	1	1	1	1	1	1	1	1	1	1	1	24
264	1	1	1	1	1	1	1	1	1	1	1	1	1	1	1	1	1	1	1	1	1	1	1	1	24
265	1	1	1	1	1	1	1	1	1	1	1	1	1	1	1	1	1	1	1	1	1	1	1	1	24
266	1	1	1	1	1	1	1	1	1	1	1	1	1	1	1	1	1	1	1	1	1	1	1	1	24
267	1	1	1	1	1	1	1	1	1	1	1	1	1	1	1	1	1	1	1	1	1	1	1	1	24
268	1			1	1	1	1	1	1	1	1	1	1	1	1	1	1	1	1	1			1	1	20
269	1			1	1	1	1	1	1	1	1	1	1	1	1	1	1	1	1	1			1	1	20
270	1	1	1	1	1	1	1	1	1	1	1	1	1	1	1	1	1		1	1	1		1	1	22
271	1	1	1	1	1	1	1	1	1	1	1	1	1	1	1	1	1	1	1	1	1		1	1	23
272	1	1	1	1	1	1	1	1	1	1	1	1	1	1	1	1	1	1	1	1	1	1	1	1	24
273	1	1	1	1	1	1	1	1	1	1	1	1	1	1	1	1	1	1	1	1	1	1	1	1	24
274	1	1	1	1	1	1	1	1	1	1	1	1	1	1	1	1	1			1	1		1	1	21
275	1			1	1	1	1	1	1	1	1	1	1	1	1	1	1	1	1	1			1	1	20
276	1	1	1		1	1	1	1	1	1	1	1	1	1	1	1	1	1	1	1	1	1	1	1	23
277	1	1	1	1	1	1	1	1	1	1	1	1	1	1	1	1	1	1	1	1	1	1	1	1	24
278	1	1	1	1	1	1	1	1	1	1	1	1	1	1	1	1	1	1	1	1	1	1	1	1	24
279	1	1	1	1	1	1	1	1	1	1	1	1	1	1	1	1	1	1	1	1	1	1	1	1	24
280	1			1	1	1	1	1	1	1	1	1	1	1	1	1	1	1	1	1		1	1	1	21
281	1	1	1	1	1	1	1	1	1	1	1	1	1	1	1	1	1	1	1	1	1	1	1	1	24
282	1	1	1	1	1	1	1	1	1	1	1	1	1	1	1	1	1	1	1	1	1	1	1	1	24
283	1	1	1	1	1	1	1	1	1	1	1	1	1	1	1	1	1	1	1	1	1	1	1	1	24
284	1				1	1			1	1	1	1		1	1	1	1	1	1	1		1	1	1	17
285	1	1	1	1	1	1	1	1	1	1	1	1	1	1	1	1	1	1	1	1	1	1	1	1	24
286		1	1	1	1	1	1	1	1	1	1	1	1	1	1	1	1	1		1	1	1	1	1	22
287		1		1	1	1	1	1	1	1	1	1	1	1	1	1	1	1	1	1	1	1	1	1	22
288	1	1	1	1	1	1	1	1	1	1	1	1	1	1	1	1	1	1	1	1	1	1	1	1	24
289	1	1	1	1	1	1	1	1	1	1	1	1	1	1	1	1	1	1	1	1	1	1	1	1	24
290	1	1	1	1	1	1	1	1	1	1	1	1	1	1	1	1	1	1	1	1	1	1	1	1	24
291	1			1	1	1	1	1	1	1	1	1	1	1	1	1	1	1	1	1			1	1	20
292	1	1	1	1	1	1	1	1	1	1	1	1	1	1	1	1	1	1	1	1	1	1	1	1	24
293	1									1	1	1	1	1	1	1	1	1	1	1		1	1	1	15
294	1			1	1	1	1	1	1	1	1	1		1	1	1	1	1	1	1			1	1	19
295	1			1	1	1	1	1	1	1	1	1	1	1	1	1	1	1	1	1		1	1	1	21
296	1			1	1	1	1	1	1			1	1	1	1	1	1			1			1		15
297	1	1	1	1	1	1	1	1	1	1	1	1	1	1	1	1	1	1	1	1	1	1	1		23
298	1	1	1			1	1	1	1	1	1	1		1	1	1	1	1	1	1	1		1	1	20
299	1	1	1	1	1	1	1	1	1	1	1	1	1	1	1	1	1	1	1	1	1	1	1	1	24
302	1	1	1	1	1	1	1	1	1	1	1	1	1	1	1	1	1			1	1		1	1	21
303	1	1	1	1	1	1	1	1	1	1	1	1	1	1	1	1	1	1	1	1	1	1	1	1	24
304	1			1	1	1	1	1	1	1	1	1	1	1	1	1	1	1	1	1		1	1	1	21
305	1	1								1	1	1	1	1	1	1	1	1	1	1			1	1	15
306	1	1	1	1	1	1	1	1	1	1	1	1	1	1	1	1	1	1	1	1	1	1	1	1	24
307	1			1	1	1	1	1	1	1	1	1	1	1	1	1	1	1	1	1			1	1	20
308	1	1	1	1	1	1	1	1	1	1	1	1	1	1	1	1	1	1	1	1	1	1	1	1	24
309	1			1	1	1	1	1	1	1	1	1	1	1	1	1	1	1	1	1		1	1	1	21
Total	**296**	**238**	**232**	**280**	**297**	**295**	**295**	**297**	**293**	**286**	**297**	**304**	**300**	**304**	**305**	**304**	**305**	**278**	**265**	**303**	**234**	**223**	**296**	**275**	6802

Supplementary Table S5. Relative propensity for biofilm formation in *Salmonella* spp. strains.

Strain code	Average OD±SD	Biofilm formation	ARGs/VMs
83	0.767±0.21	weak	-

192	0.89±0.37	weak	-
204	0.476±0.18	weak	-
205	0.439±0.08	weak	-
206	0.473±0.04	weak	
216	0.654±0.41	weak	-
217	0.700±0.40	weak	+
218	0.565±0.19	weak	-
228	0.494±0.06	weak	-
240	0.556±0.49	weak	-
241	0.455±0.74	weak	+
242	0.497±0.49	weak	+
245	0.454±0.09	weak	-
264	0.932±0.67	moderate	-
265	0.527±0.10	weak	-
266	0.508±0.05	weak	-
267	0.465±0.06	weak	-

Supplementary Table S6. Potentially significant correlations found between ARGs and VMs present in the samples.

<table>
<tr><th>ARGs</th><th>Virulence markers</th><th>p-value</th></tr>
<tr><td>tet(A)</td><td rowspan="5">spvC</td><td><0.001</td></tr>
<tr><td>sul 1</td><td><0.05</td></tr>
<tr><td>qnrB</td><td><0.05</td></tr>
<tr><td>bla_{TEM}</td><td><0.001</td></tr>
<tr><td>bla_{SHV}</td><td><0.05</td></tr>
</table>

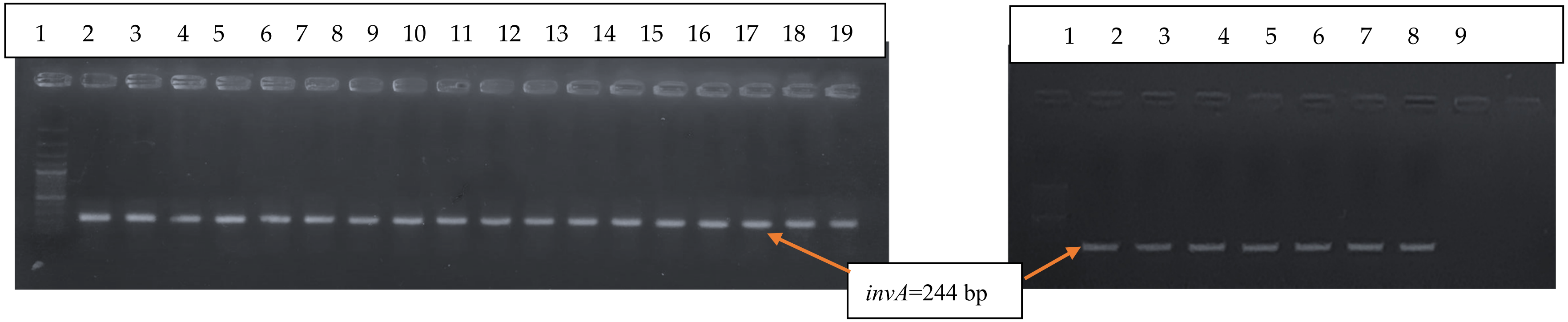

Figure S1. Electrophoresis gel for *invA* gene detection in selected *Salmonella* spp. strains. Lines: 1-Molecular Size Marker (ThermoScientific, 1500 bp, 2-strain 58, 3-59, 4-60, 5-61,6 -62, 7-63, 8-64, 9-65, 10-66, 11-67, 12-68, 13-69, 14-70, 15-71, 16-72, 17-73, 18-74, 19-*Salmonella* positive control. Positives strains: 58;59;60;61;62;63;64;65;66;67;68;69;70;71;72;73;74;75.

Figure S2. Electrophoresis gel for *invA* gene detection in selected *Salmonella* spp. strains. Lines: 1-Molecular Size Marker (ThermoScientific, 1500 bp, 2-strain 303, 3-304, 4-305 ,5-306, 6-307, 7-308, 8-309, 9- *S. Typhimurium* 14028, negative control. Positives strains: 303;304;305;306;307;308;309.

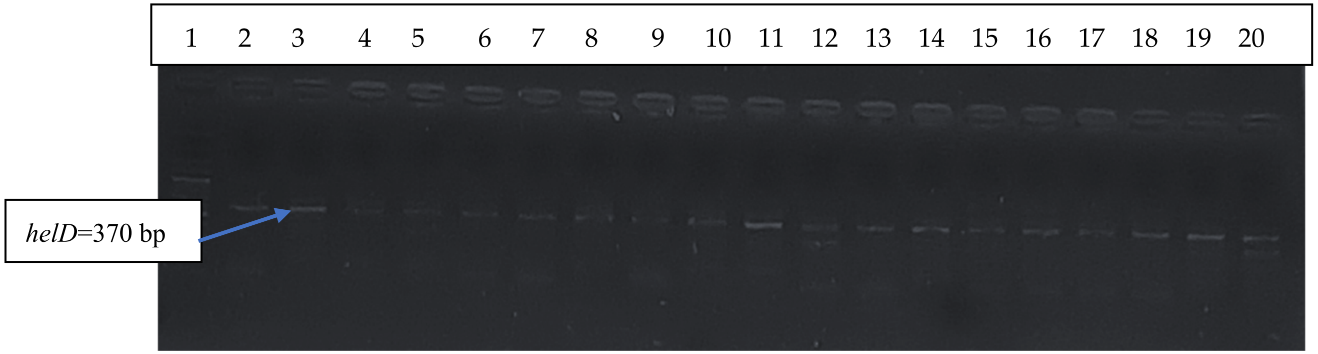

Figure S3. Electrophoresis gel for *pldA* and *helD* genes detection in selected *Salmonella* spp. strains. Lines: 1-Molecular Size Marker (ThermoScientific, 1500 bp, 2-strain 76, 3-77, 4-78, 5-79, 6-80, 7-81, 8-82, 9-83, 10-84, 11-85, 12-86, 13-87, 14-88, 15-89, 16-90, 17-91, 18-92, 19-93, 20-positive control. Positives strains for *helD* gene: 76, 77, 78, 79, 80, 81, 82, 83, 84, 85, 86, 87, 88, 89, 90, 91, 92, 93.

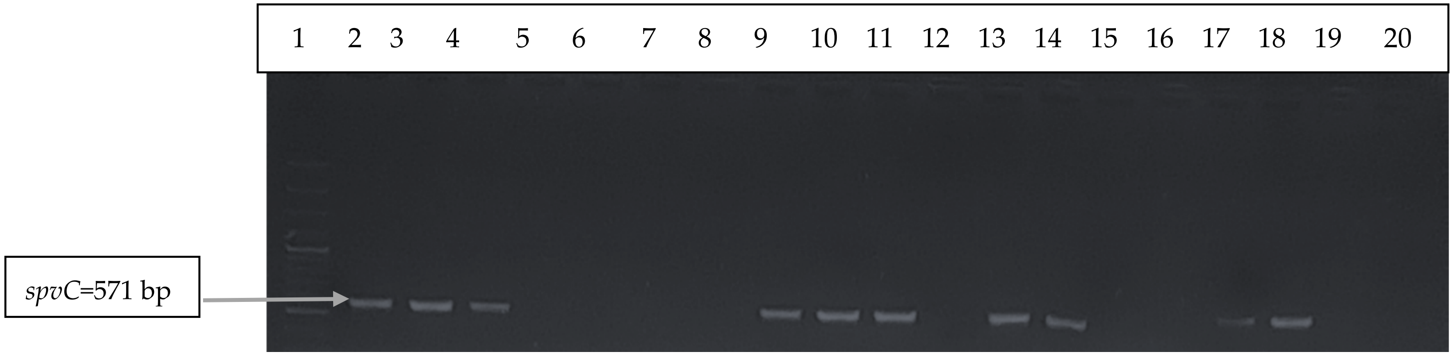

Figure S4. Electrophoresis gel for *spvC* gene detection in selected *Salmonella* spp. strains. Lines: 1-Molecular Size Marker (ThermoScientific, 1500 bp, 2-strain 152, 3-153, 4-154, 5-155, 6-156, 7-157, 8-158, 9-159, 10-160, 11-161, 12-162, 13-163, 14-164, 15-165, 17-167, 18-168, 19-170, 20- *S. Typhimurium* 14028, negative control. Positives strains: 152;153;154;159;160;161;163;164;167;168.

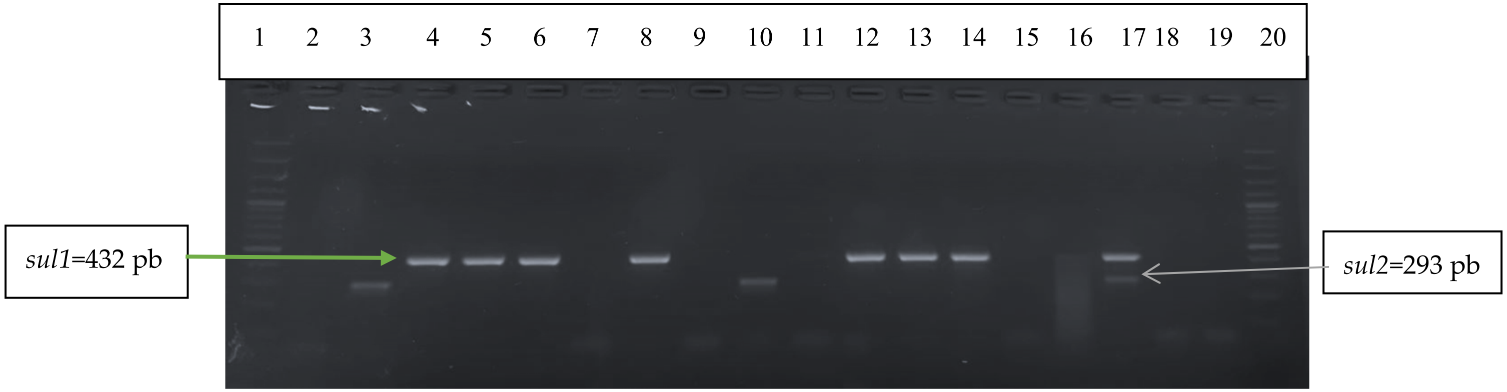

Figure S5. Electrophoresis gel for *sul1* and *sul2* genes detection in selected *Salmonella* spp. strains. Lines: 1-Molecular Size Marker (ThermoScientific, 1500 bp, 2-strain 129, 3-132, 4-146, 5-148, 6-152, 7-170, 8-171, 9-175, 10-176, 11-190, 12-200, 13-207, 14-230, 15-234, 16-256, 17-296, 18-297, 19- *S. Typhimurium* 14028, negative control, 20-Molecular Size Marker. Positives strains: *sul1* gene: 146; 148; 152; 171; 200; 207; 230 and *sul2*: 132; 176; 296.

1 2 3 4 5 6 7 8 9 10 11 12 13 14 15 16 17 18 19 20

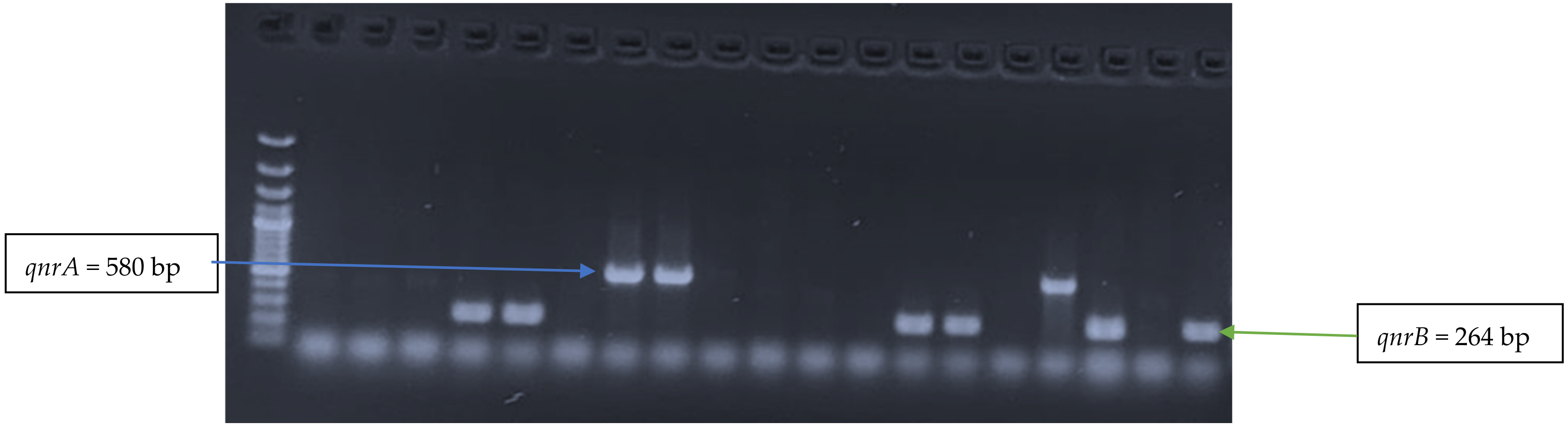

Figure S6. Electrophoresis gel for *qnrA* and *qnrB* genes detection in selected *Salmonella* spp. strains. Lines: 1-Molecular Size Marker (ThermoScientific, 1500 bp, 2-strain 113, 3-121, 4-133, 5-135, 6-136, 7-137, 8-146, 9-148, 10-171, 11-196, 12-200, 13-207, 14-242, 15-252, 16-254, 17-256, 18-274, 19-*S. Typhimurium* 14028, negative control, 20-302. Positives strains: *qnrA* gene: 146; 148; 256 and *qnrB*: 135; 136; 242; 252; 274; 302.

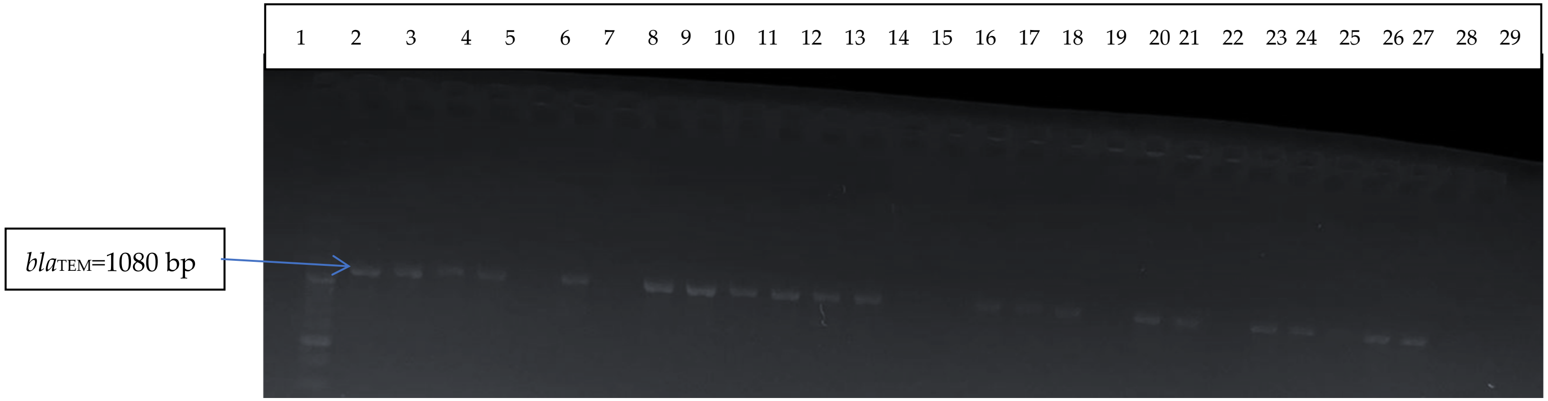

Figure S7. Electrophoresis gel for *bla*TEM and *bla*KPC genes detection in selected *Salmonella* spp. strains. Lines: 1-Molecular Size Marker (ThermoScientific, 1500 bp, 2-strain 9, 3-10, 4-15, 5-18, 6-21, 7-34, 8-41, 9-58, 10-61, 11-63, 12-67, 13-69, 14-73, 15-75, 16-80, 17-82, 18-85, 19-88, 20-93, 21-96, 22-97, 23-101, 24-103, 25-107, 26-109, 27-111, 28-118, 29-123, 30-124. Positives strains: *bla*TEM: 9;10;15;18;34;58;61;63;67;69;73;82;85;88;96;97;103;107;109;111;118.

Supplementary Table S7. The diverse virulence strategies of *S. enterica* clones, particularly the widespread ST19 clone, in outpatient infections.

					Adherence	Effector delivery system	Metabolic factors	Stress survival	Antimicrobial activity/Competitive advantage	Regulation	Immune modulation
19	10	Typhimurium	TEM-1	**10/10**; IncB/O/K/Z_2, IncFIB(S)_1, IncFII(S)_1, IncX3_1, IncX1_1, ColRNAI_1, Col440I_1, IncFIC(FII)_1, IncR_1, IncFIB(AP001918)_1	*fimI, fimC, fimD, fimH, fimF, csgC, csgA, csgB, csgD, csgE, csgF, csgG, sinH, ratB, lpfE, lpfD, lpfC, lpfB, lpfA,misL, ompA*	*invH, invF, invG, invE, invA, invB, invC, invI, invJ, sopD, ssaU, ssaT, ssaS, ssaR, ssaQ, ssaP, ssaO, ssaN, ssaV, ssaM, ssaL, ssaK, ssaJ, ssaI, ssaH, ssaG, sseG, sseF, sscB, sseE, sseD, sseC, sscA, sseB, sseA, ssaE, ssaD, ssaC, avrA, sseL, sseK2, sseI/srfH, slrP, sseK1, sopD2, sopA, sopE2, steC, sseJ, steB, sifB, steA, sicA, sipB/sspB, sipC/sspC, sipD, sipA/sspA, sicP, gogB, spaO, spaP, spaQ, spaR, spaS, sopB/sigD, orgA, orgB, orgC, pipB, pipB2, prgH, prgI, prgJ, prgK, sptP, sifA, sifA, pipB2, spvC, B, R*	*entB, entA, mgtB, mgtC, fepC, fepG,*	*sodCI*	*mig-14*	*spiC/ssaB*	*rck*

34	5	Typhimurium	TEM-1	**2/5**; Col(VCM04)_1	*fimI, fimC, fimD, fimH, fimF, csgC, csgA, csgB, csgD, csgE, csgF, csgG, sinH, ratB, lpfE, lpfD, lpfC, lpfB, lpfA,misL, ompA*	*invH, invF, invG, invE, invA, invB, invC, invI, invJ, sopD, ssaU, ssaT, ssaS, ssaR, ssaQ, ssaP, ssaO, ssaN, ssaV, ssaM, ssaL, ssaK, ssaJ, ssaI, ssaH, ssaG, sseG, sseF, sscB, sseE, sseD, sseC, sscA, sseB, sseA, ssaE, ssaD, ssaC, avrA, sseL, sseK2, sseI/srfH, slrP, sseK1, sopD2, sopA, sopE2, steC, sseJ, steB, sifB, steA, sicA, sipB/sspB, sipC/sspC, sipD, sipA/sspA, sicP, gogB, spaO, spaP, spaQ, spaR, spaS, sopB/sigD, orgA, orgB, orgC, pipB, pipB2, prgH, prgI, prgJ, prgK, sptP, sifA, sifA, pipB2*	*entB, entA, mgtB, mgtC, fepC, fepG,*	*sodCI*	*mig-14*	*spiC/ssaB*

33	3	Hadar	3/3; Col8282_1, Col440I_1	*fimI, fimC, fimD, fimH, fimF, csgC, csgA, csgB, csgD, csgE, csgF, csgG, sinH, ratB, lpfE, lpfD, lpfC, lpfB, lpfA,misL, ompA*	*invH, invF, invG, invE, invA, invB, invC, invI, invJ, sopD, ssaU, ssaT, ssaS, ssaR, ssaQ, ssaP, ssaO, ssaN, ssaV, ssaM, ssaL, ssaK, ssaJ, ssaI, ssaH, ssaG, sseG, sseF, sscB, sseE, sseD, sseC, sscA, sseB, sseA, ssaE, ssaD, ssaC, avrA, sseL, sseK2, sseI/srfH, slrP, sseK1, sopD2, sopA, sopE2, steC, sseJ, steB, sifB, steA, sicA, sipB/sspB, sipC/sspC, sipD, sipA/sspA, sicP, gogB, spaO, spaP, spaQ, spaR, spaS, sopB/sigD, orgA, orgB, orgC, pipB, pipB2, prgH, prgI, prgJ, prgK, sptP, sifA, sifA, pipB2*	*entB, entA, mgtB, mgtC, fepC, fepG*	*sodCI*	*mig-14*	*spiC/ssaB*

32	1	Infantis	IncI1_1_Alpha	*fimI, fimC, fimD, fimH, fimF, csgC, csgA, csgB, csgD, csgE, csgF, csgG, sinH, ratB, lpfE, lpfD, lpfC, lpfB, lpfA,misL, ompA*	*invH, invF, invG, invE, invA, invB, invC, invI, invJ, sopD, ssaU, ssaT, ssaS, ssaR, ssaQ, ssaP, ssaO, ssaN, ssaV, ssaM, ssaL, ssaK, ssaJ, ssaI, ssaH, ssaG, sseG, sseF, sscB, sseE, sseD, sseC, sscA, sseB, sseA, ssaE, ssaD, ssaC, avrA, sseL, sseK2, sseI/srfH, slrP, sseK1, sopD2, sopA, sopE2, steC, sseJ, steB, sifB, steA, sicA, sipB/sspB, sipC/sspC, sipD, sipA/sspA, sicP, gogB, spaO, spaP, spaQ, spaR, spaS, sopB/sigD, orgA, orgB, orgC, pipB, pipB2, prgH, prgI, prgJ, prgK, sptP, sifA, sifA, pipB2*	*entB, entA, mgtB, mgtC, fepC, fepG*	*sodCI*	*mig-14*	*spiC/ssaB*

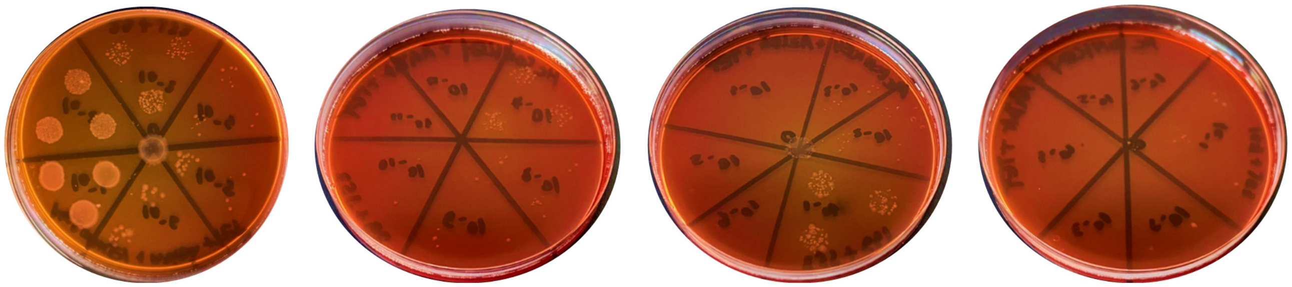

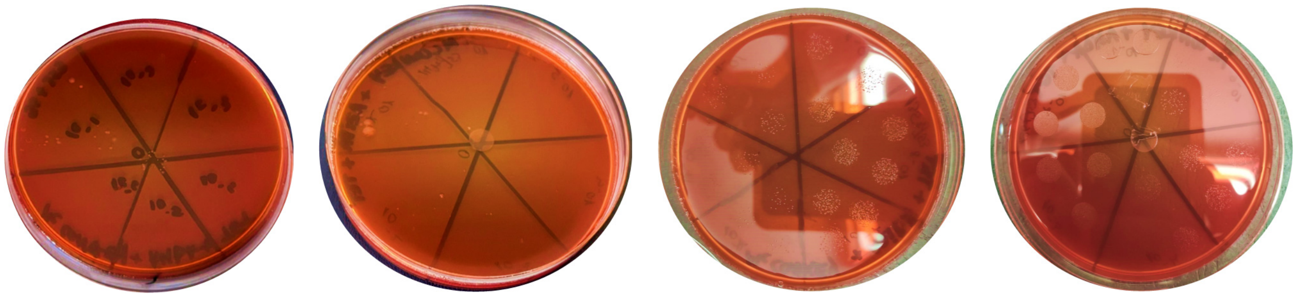

Figure S8. Transconjugants colony forming units aspect (strains encoded *S. enterica* 29 + J53; 149 + J53; 107 + J53; 96 + J53; 147 + J53; 135 + J53; 136 + J53) on MacConkey agar supplementyed with sodium azide (150 mg/L) and tetracycline (8 mg/L).

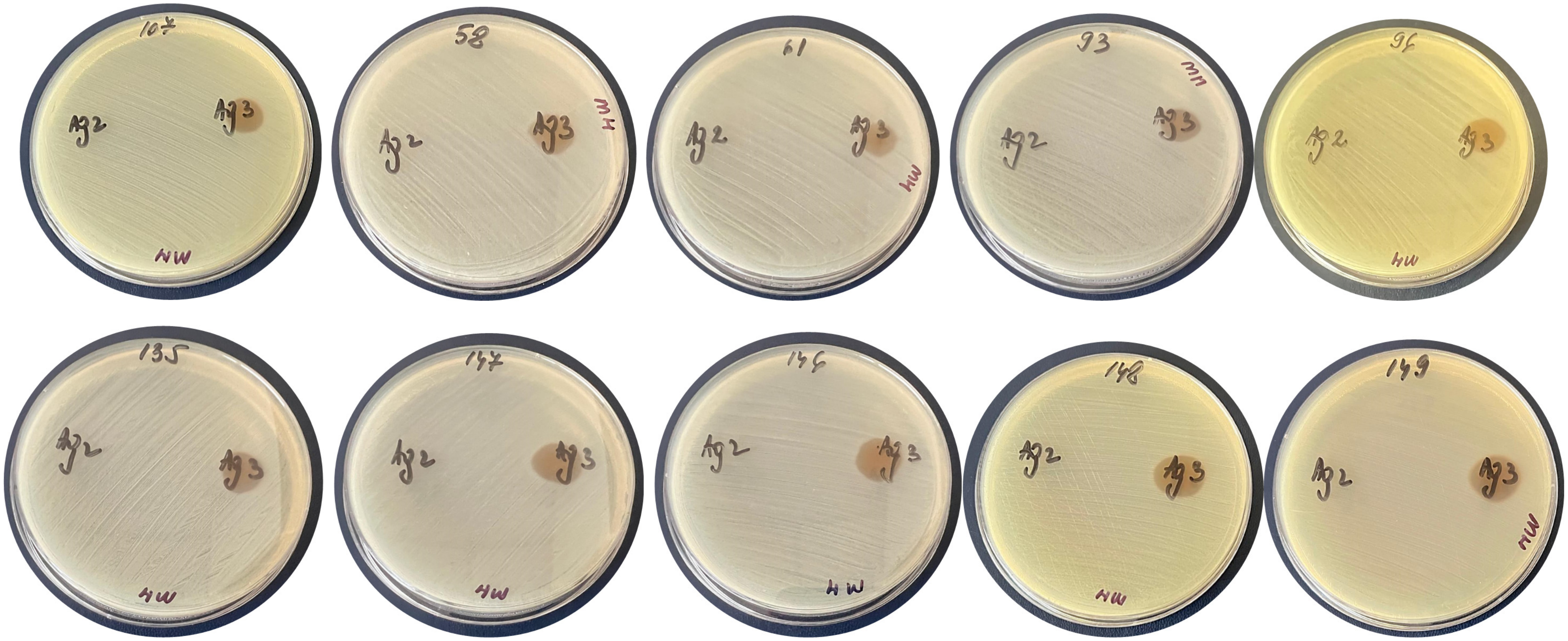

Figure S9. Disk diffusion screening assays of the antimicrobial activity of the Ag2NP and Ag3NPsol against *Salmonella enterica* strains encoded 107, 58, 61, 93, 96, 135, 147, 146, 148 and 149 isolated from stool pediatric patients.

Supplementary Table S8. The diameter of the inhibition zone of AgNPsol against *Salmonella* spp. strains.

**Strain code**	**Clone**	**Serotype**	**Patient age**	**Inhibition zone (mm)**	**AU**
149	ST34	*S*. 4,[5],12:i:-	4 years old	15	2
152	ST19	*S*. Typhimurium	2 years old	0	0
18	ST19	*S*. Typhimurium	4 years old	0	0
21	ST19	*S*. Typhimurium	2 years old	0	0
29	ST33	*S*. Hadar	1 years old	0	0
34	ST19	*S*. Typhimurium	1 years old	0	0
58	ST19	*S*. Typhimurium	4 years old	12	2
61	ST19	*S*. Typhimurium	3 years old	12	2
93	ST32	*S*. Infantis	1 years old	11	2
96	ST34	*S*. 4,[5],12:i:-	6 years old	13	2
106	ST19	*S*. Typhimurium	2 years old	0	0
107	ST34	*S*. 4,[5],12:i:-	4 years old	11	2
118	ST34	*S*. 4,[5],12:i:-	2 years old	0	0
129	ST19	*S*. Typhimurium	6 years old	0	0
135	ST33	*S*. Hadar	2 months old	11	2
136	ST33	*S*. Hadar	2 years old	0	0
146	ST19	*S*. Typhimurium	2 years old	12	2
147	ST34	*S*. 4,[5],12:i:-	9 months old	14	2
148	ST19	*S*. Typhimurium	1 years old	15	2
ATCC 14028		*S*. Typhimurium	-	0	0

Supplementary Table S9. The MIC and the corresponding MIC/2 and MIC/4 values of AgNPsol for PICA determination in the selected *S. enterica* strains.

Strain code	Clone	Serotype	MIC (µg/mL)	MIC/2 (µg /mL)	PICA%	p-value	MIC/4 (µg /mL)	PICA%	*p*-value
149	ST34	*S*. 4,[5],12:i:-	104,17	52,08	62,22%	0,5762	26,04	144,29%	0,4755
152	ST19	*S*. Typhimurium	31,25	15,63	168,51%	0,1916	7,81	101,61%	0,999
18	ST19	*S*. Typhimurium	62,50	31,25	156,93%	0,3067	15,63	158,56%	0,2883
21	ST19	*S*. Typhimurium	500	250	1,81%	0,0436	125	0,71%	**0,041**

29	ST33	*S*. Hadar	312,50	156,25	9,56%	0,0666	78,13	55,73%	0,4757
34	ST19	*S*. Typhimurium	500	250	20,14%	0,1138	125	2,49%	0,0453
58	ST19	*S*. Typhimurium	23,43	11,71	45,49%	0,3357	5,86	74,61%	0,7733
61	ST19	*S*. Typhimurium	125	62,50	68,52%	0,6773	31,25	133,62%	0,6428
93	ST32	*S*. Infantis	46,88	23,44	78,90%	0,8358	11,72	87,29%	0,9362
96	ST34	*S*. 4,[5],12:i:-	125	62,50	175,72%	0,1386	31,25	368,16%	**<0,0001**
106	ST19	*S*. Typhimurium	500	250	2,67%	**0,0458**	125	2,16%	0,0445
107	ST34	*S*. 4,[5],12:i:-	62,50	31,25	54,71%	0,4607	15,63	110,33%	0,9572
118	ST34	*S*. 4,[5],12:i:-	62,50	31,25	39,29%	0,2649	15,63	137,19%	0,5856
129	ST19	*S*. Typhimurium	500	250	16,14%	0,0935	125	4,75%	**0,0514**
135	ST33	*S*. Hadar	62,50	31,25	64,06%	0,6056	15,63	121,82%	0,8257
136	ST33	*S*. Hadar	500	250	1,49%	0,0429	125	1,20%	**0,0422**
146	ST19	*S*. Typhimurium	46,88	23,44	31,83%	0,1944	11,72	92,93%	0,9797
147	ST34	*S*. 4,[5],12:i:-	104,17	52,08	93,06%	0,9804	26,04	188,86%	0,0724
148	ST19	*S*. Typhimurium	83,33	41,67	105,56%	0,9874	20,83	155,69%	0,3213
ATCC 14028		*S*. Typhimurium	46,88	23,44	52,68%	0,4313	11,72	111,43%	0,9479
